# Supplementary material for: Bioactive Molecules from Mangrove Streptomyces qinglanensis 172205
Source: Mar Drugs. 2020 May 13;18(5):255. doi: 10.3390/md18050255 (PMC7281499; doi:10.3390/md18050255)
Supplement: Supplementary file 1 [file marinedrugs-18-00255-s001.pdf]

Supplementary Materials

Bioactive Molecules from Mangrove *Streptomyces qinglanensis* 172205

Dongbo Xu [1], Erli Tian [1], Fandong Kong [2] and Kui Hong [1,*]

[1] Key Laboratory of Combinatorial Biosynthesis and Drug Discovery, Ministry of Education, School of Pharmaceutical Sciences, Wuhan University, Wuhan 430071, China; xudongbo1990@gmail.com (D.X.); erlitian@whu.edu.cn (E.T.)

[2] Institute of Tropical Bioscience and Biotechnology, Chinese Academy of Tropical Agricultura Sciences, Haikou, Hainan, 571101, China; kongfandong@itbb.org.cn (F.K.)

* Correspondence: kuihong31@whu.edu.cn (K.H.); Tel.: +86-27-6875-2442 (K.H.)

List of Supporting Information

Figure S1. Confirmation of enterocin gene cluster disruption by PCR.

Table S1. Oligonucleotide primers used in this study.

Table S2. Deduced functions of ORFs in xan_q biosynthetic pathway of strain 172205.

Figure S2. HERESIMS spectrum of 15*R*-17,18-dehydroxantholipin (**1**).

Figure S3. IR spectrum of 15*R*-17,18-dehydroxantholipin (**1**).

Figure S4. ^{1}H NMR Spectrum (800 MHz, DMSO-d_6) of 15*R*-17,18-dehydroxantholipin (**1**).

Figure S5. ^{13}C NMR Spectrum (200 MHz, DMSO-d_6) of 15*R*-17,18-dehydroxantholipin (**1**).

Figure S6. COSY Spectrum of 15*R*-17,18-dehydroxantholipin (**1**).

Figure S7. HSQC Spectrum of 15*R*-17,18-dehydroxantholipin (**1**).

Figure S8. HMBC Spectrum of 15*R*-17,18-dehydroxantholipin (**1**).

Figure S9. HERESIMS spectrum of (3*E*,5*E*,7*E*)-3-methyldeca-3,5,7-triene-2,9-dione (**2**).

Figure S10. IR spectrum of (3*E*,5*E*,7*E*)-3-methyldeca-3,5,7-triene-2,9-dione (**2**).

Figure S11. ^{1}H NMR Spectrum (500 MHz, CD_3OD-d_4) of (3*E*,5*E*,7*E*)-3-methyldeca-3,5,7-triene-2,9-dione (**2**).

Figure S12. ^{13}C NMR Spectrum (125 MHz, CD_3OD-d_4) of (3*E*,5*E*,7*E*)-3-methyldeca-3,5,7-triene-2,9-dione (**2**).

Figure S13. COSY Spectrum of (3*E*,5*E*,7*E*)-3-methyldeca-3,5,7-triene-2,9-dione (**2**).

Figure S14. HSQC Spectrum of (3*E*,5*E*,7*E*)-3-methyldeca-3,5,7-triene-2,9-dione (**2**).

Figure S15. HMBC Spectrum of (3*E*,5*E*,7*E*)-3-methyldeca-3,5,7-triene-2,9-dione (**2**).

Figure S16. ROESY Spectrum of (3*E*,5*E*,7*E*)-3-methyldeca-3,5,7-triene-2,9-dione (**2**).

Figure S17. HERESIMS spectrum of qinlactone A (**3**).

Figure S18. IR spectrum of qinlactone A (**3**).

Figure S19. ^{1}H NMR Spectrum (500 MHz, CD_3OD-d_4) of qinlactone A (**3**).

Figure S20. ^{13}C NMR Spectrum (125 MHz, CD_3OD-d_4) of qinlactone A (**3**).

Figure S21. COSY Spectrum of qinlactone A (**3**).

Figure S22. HSQC Spectrum of qinlactone A (**3**).

Figure S23. HMBC Spectrum of qinlactone A (**3**).

Figure S24. ROESY Spectrum of qinlactone A (**3**).

Figure S25. HERESIMS spectrum of qinlactone B (**4**).

Figure S26. IR spectrum of qinlactone B (**4**).

Figure S27. ^{1}H NMR Spectrum (500 MHz, CD_3OD-d_4) of qinlactone B (**4**).

Figure S28. ^{13}C NMR Spectrum (125 MHz, CD_3OD-d_4) of qinlactone B (**4**).

Figure S29. COSY Spectrum of qinlactone B (**4**).

Figure S30. HSQC Spectrum of qinlactone B (**4**).

Figure S31. HMBC Spectrum of qinlactone B (**4**).

Figure S32. ROESY Spectrum of qinlactone B (**4**).

Figure S33. HERESIMS spectrum of qinlactone C (**5**).

Figure S34. IR spectrum of qinlactone C (**5**).

Figure S35. ^{1}H NMR Spectrum (500 MHz, CD_3OD-d_4) of qinlactone C (**5**).

Figure S36. ^{13}C NMR Spectrum (125 MHz, CD_3OD-d_4) of qinlactone C (**5**).

Figure S37. COSY Spectrum of qinlactone C (**5**).

Figure S38. HSQC Spectrum of qinlactone C (**5**).

Figure S39. HMBC Spectrum of qinlactone C (**5**).

Figure S40. ROESY Spectrum of qinlactone C (**5**).

Figure S41. Comparison of genetic organization of xan_q in strain 172205 and *xan* in *S. flavogriseus*.

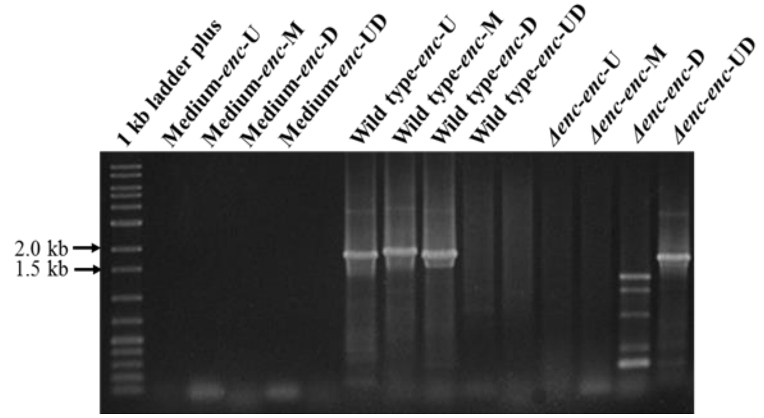

Figure S1. Confirmation of enterocin gene cluster disruption by PCR. Sample "medium" were designed as negative control.

Table S1. Oligonucleotide primers used in this study.

**Primers**	**Nucleotide sequence（5'—3'）**	**PCR product（bp）**
enc-L1	CCTAGGCAGTTCCATCACCCCGTTCG (*Avr* II)	1870
enc-L2	AAGCTTGTCGTCGCAGCAGCAGTTCG (*Hin*d III)	
enc-R1	CATATGAGAGGGCGGACGGGAACTGC (*Nde* I)	1829
enc-R2	CCTAGGGCGCCATCCCAACGGGCTAC (*Avr* II)	
enc-U1	GGTCGAGCGTGCGCTGTCCA	1821
enc-U2	CGAGACCGTGGTGCGGGAGA	
enc-UD1	CTGCTGCGCTTCGTGTTCGA	1808
enc-UD2	GGGAGCACAGCGGCTACTGG	
enc-D1	GGCCGGTCAGGGTCTTCAGT	1773
enc-D2	CACGGTCTGCGGAGGTCTCA	
enc-M1	CTCAGCAGGCGCTGGAGGGT	1885
enc-M2	GGGCTGGGTCTCGCATCTGG	

Table S2. Deduced functions of ORFs in xan_q biosynthetic pathway of strain 172205

Gene (aa)	Proposed function	Protein Homologs	Identity/Similarity (%)
orfO11 (154)	pyridoxamine 5'-phosphate oxidase	ADL35_39695, *S.* sp. NRRL WC-3753	85/94
orfS1 (434)	dehydrogenase	XanS1, *S. flavogriseus*	69/79
orfJ (171)	hypothetical protein	XanJ, *S. flavogriseus*	53/73
orfP (551)	spore coat protein	Xan, *S. flavogriseus*	67/78
orfG (398)	inactive gylcosyltransferase	XanG, *S. flavogriseus*	69/75
orfA (618)	asparagine synthase	XanA, *S. flavogriseus*	86/91
orfR1 (223)	transcriptional regulator	XanR1, *S. flavogriseus*	69/78
orfO2 (399)	cytochrome P450 hydroxylase	XanO2, *S. flavogriseus*	90/95
orfK (65)	3Fe-S ferredoxin	XanK, *S. flavogriseus*	82/90
orfI (395)	glutathionylspermidine synthase	ADL27_17915, *S.* sp. NRRL F-6602	91/95
orfJ1 (140)	hypothetical protein	ADL27_17920, *S.* sp. NRRL F-6602	68/75
orfO5 (580)	FAD-binding monooxygenase	XanO5, *S. flavogriseus*	79/87
orfM3 (338)	methyltransferase	XanM3, *S. flavogriseus*	83/88
orfO4 (544)	FAD-binding monooxygenase	XanO4, *S. flavogriseus*	85/90
orfM2 (341)	O-methyltransferase	XanM2, *S. flavogriseus*	73/81
orfZ5 (308)	Zn-binding oxidoreductase	SLNWT_5838, *S. albus* ATCC 21838	64/73
orfR2 (164)	transcriptional regulator	XanR2, *S. flavogriseus*	66/78
orfW (174)	hypothetical protein	XanW, *S. flavogriseus*	60/76
orfN (438)	membrane ion antiporter	XanN, *S. flavogriseus*	67/80
orfZ1 (288)	F420-dependent reductase	XanZ1, *S. flavogriseus*	79/85
orfM1 (348)	O-methyltransferase	XanM1, *S. flavogriseus*	71/81
orfQ (516)	peptide transporter	XanQ, *S. flavogriseus*	78/85
orfH (474)	non-heme halogenase	XanH, *S. flavogriseus*	84/92
orfL (176)	lactoylglutathione lyase	XanL, *S. flavogriseus*	56/73
orfO3 (403)	FAD-binding monooxygenase	XanO3, *S. flavogriseus*	76/86
orfU1 (729)	Membrane protein	SXIM_30770, *S. xiamenensis*	68/81
orfX (551)	Copper oxidase	ADL01_32595, *S.* sp. NRRL WC-3618	64/78
orfS2 (234)	dehydrogenase/reductase	XanS2, *S. flavogriseus*	71/79
orfV1 (310)	hypothetical protein	SIRAN5268, *S. iranensis*	49/62
orfB1 (447)	biotin carboxylase	XanB1, *S. flavogriseus*	87/94
orfB2 (184)	biotin carboxyl carrier protein	XanB2, *S. flavogriseus*	67/77
orfB3 (563)	carboxyl transferase	XanB3, *S. flavogriseus*	86/90
orfO6 (103)	monooxygenase	XanO6, *S. flavogriseus*	84/90
orfO7 (114)	monooxygenase	XanO7, *S. flavogriseus*	76/88
orfZ3 (250)	3-oxoacyl-ACP reductase	XanZ3, *S. flavogriseus*	84/90
orfO8 (153)	monooxygenase	XanO8, *S. flavogriseus*	80/84
orfC1 (173)	polyketide cyclase	XanC1, *S. flavogriseus*	75/83
orfE (434)	beta-ketoacyl synthase	XanE, *S. flavogriseus*	79/86
orfF (439)	beta-ketoacyl synthase	XanF, *S. flavogriseus*	88/94
orfC2 (144)	polyketide cyclase	XanC2, *S. flavogriseus*	84/91
orfT (125)	CurD-like protein	XanT, *S. flavogriseus*	84/89
orfO9 (482)	oxidase-like protein	XanO9, *S. flavogriseus*	65/78
orfO10 (153)	monooxygenase	XanO10, *S. flavogriseus*	86/91
orfV (126)	hypothetical protein	XanV, *S. flavogriseus*	89/92
orfZ4 (258)	3-oxoacyl-ACP reductase	XanZ4, *S. flavogriseus*	69/75
orfD (86)	ACP	XanD, *S. flavogriseus*	78/89
orfC3 (112)	polyketide cyclase	XanC3, *S. flavogriseus*	83/92

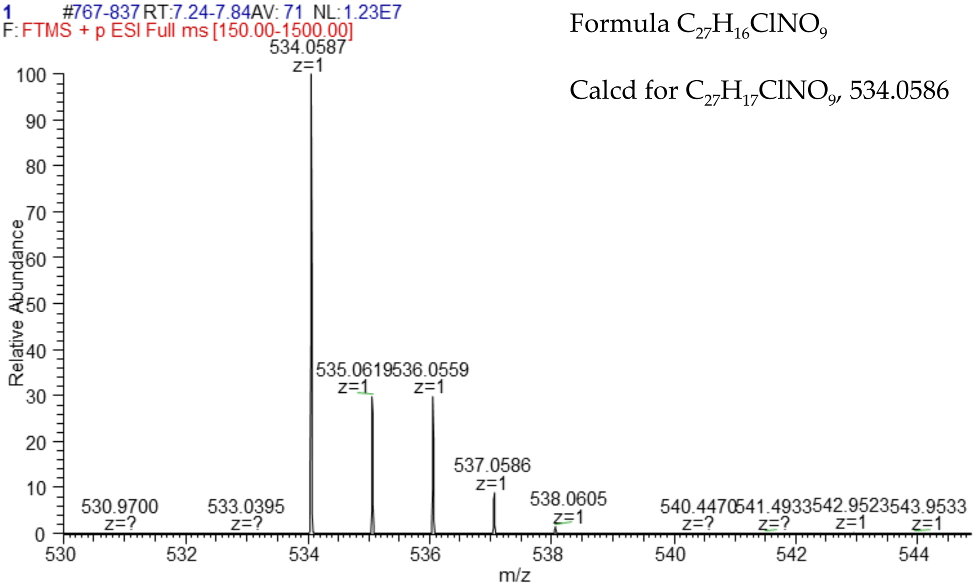

Figure S2. HERESIMS spectrum of 15*R*-17,18-dehydroxantholipin (**1**).

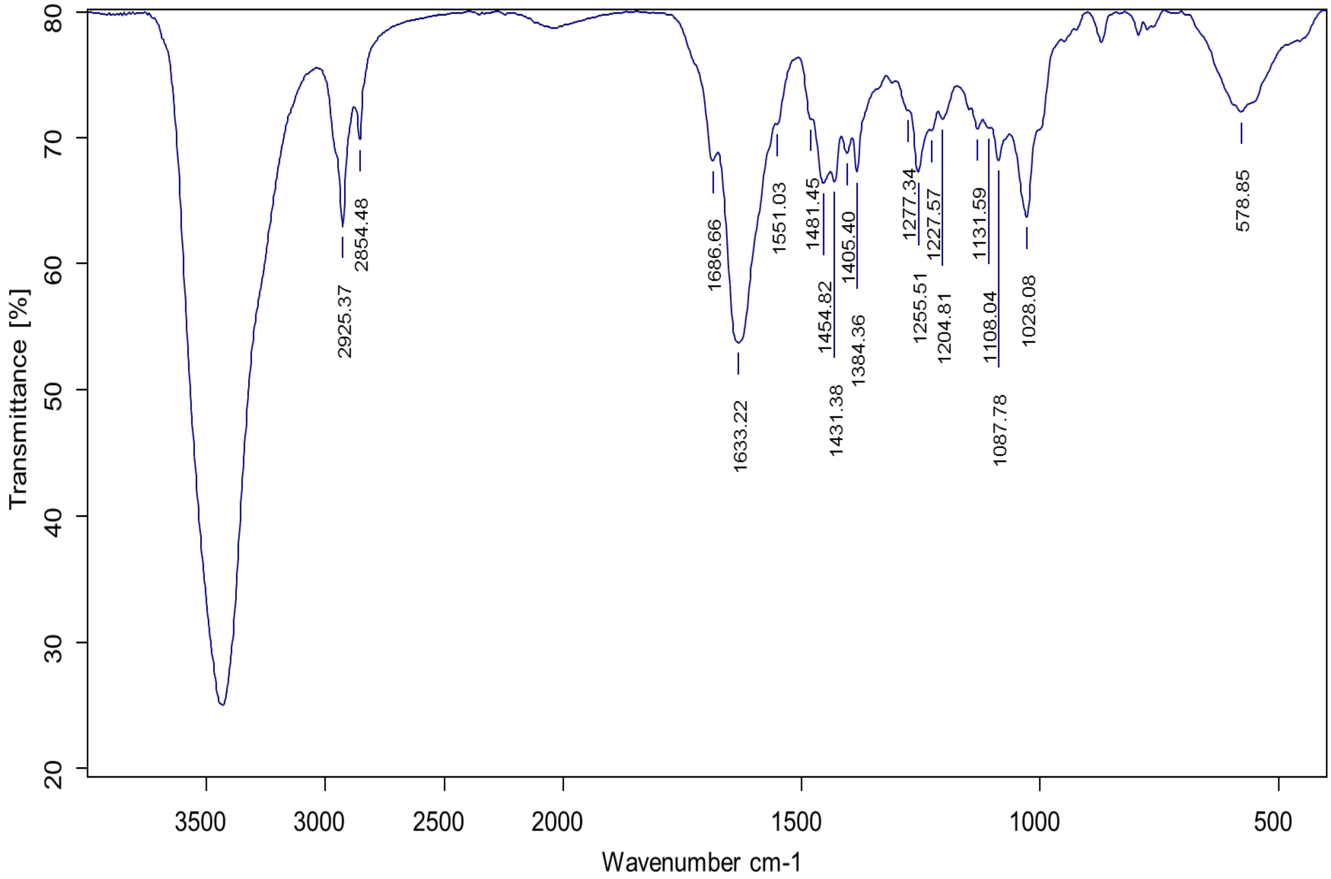

Figure S3. IR spectrum of 15*R*-17,18-dehydroxantholipin (**1**).

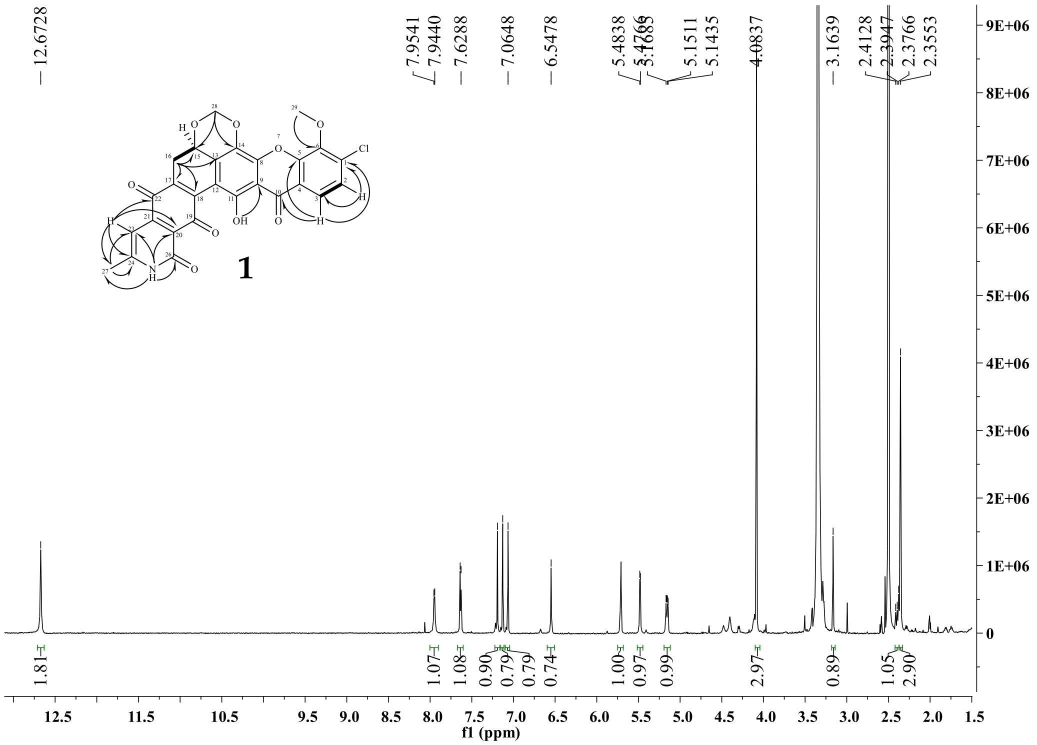

Figure S4. ^{1}H NMR Spectrum (800 MHz, DMSO-d_6) of 15*R*-17,18-dehydroxantholipin (**1**).

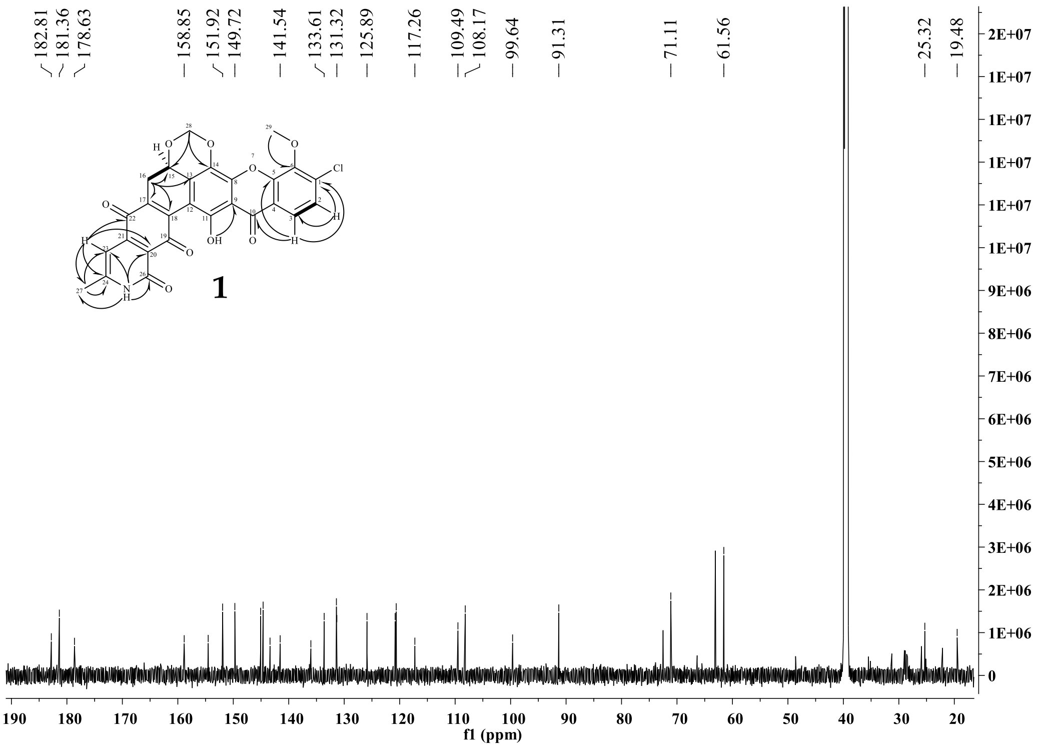

Figure S5. ^{13}C NMR Spectrum (200 MHz, DMSO-d_6) of 15*R*-17,18-dehydroxantholipin (**1**).

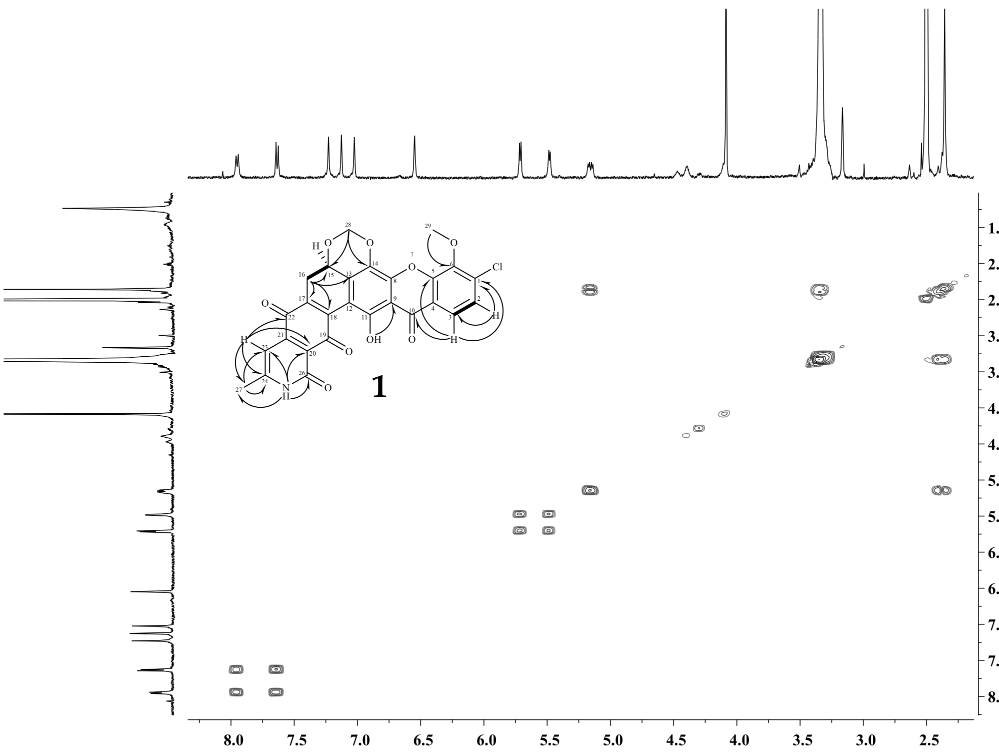

Figure S6. COSY Spectrum of 15*R*-17,18-dehydroxantholipin (**1**).

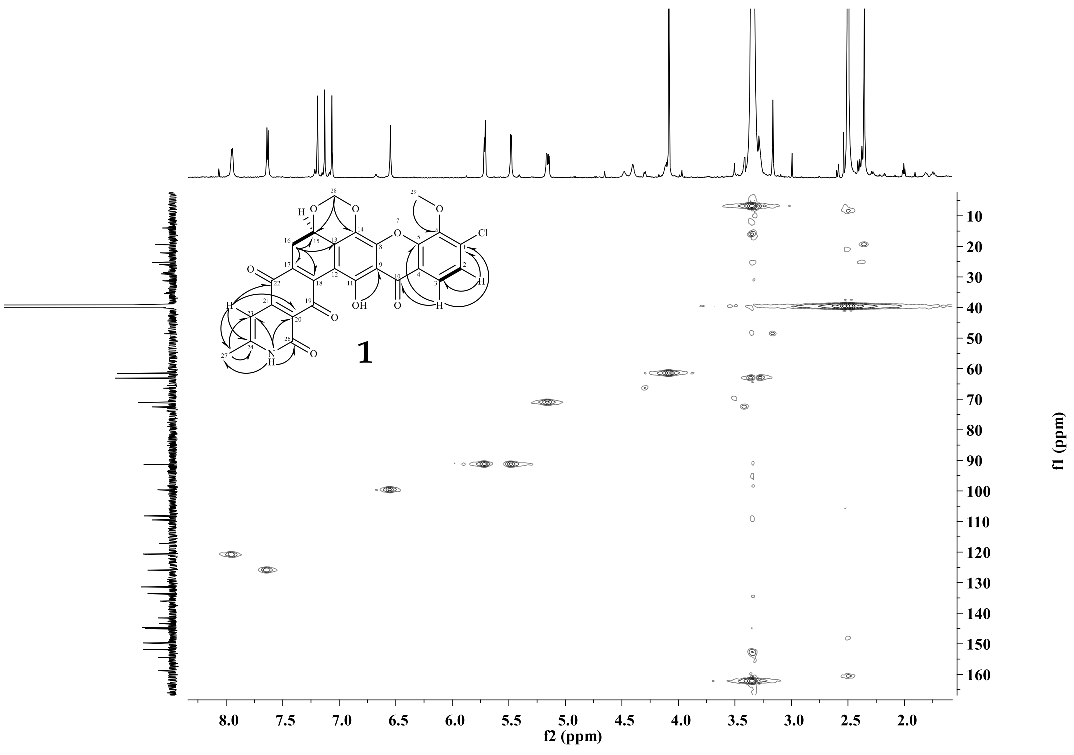

Figure S7. HSQC Spectrum of 15*R*-17,18-dehydroxantholipin (**1**).

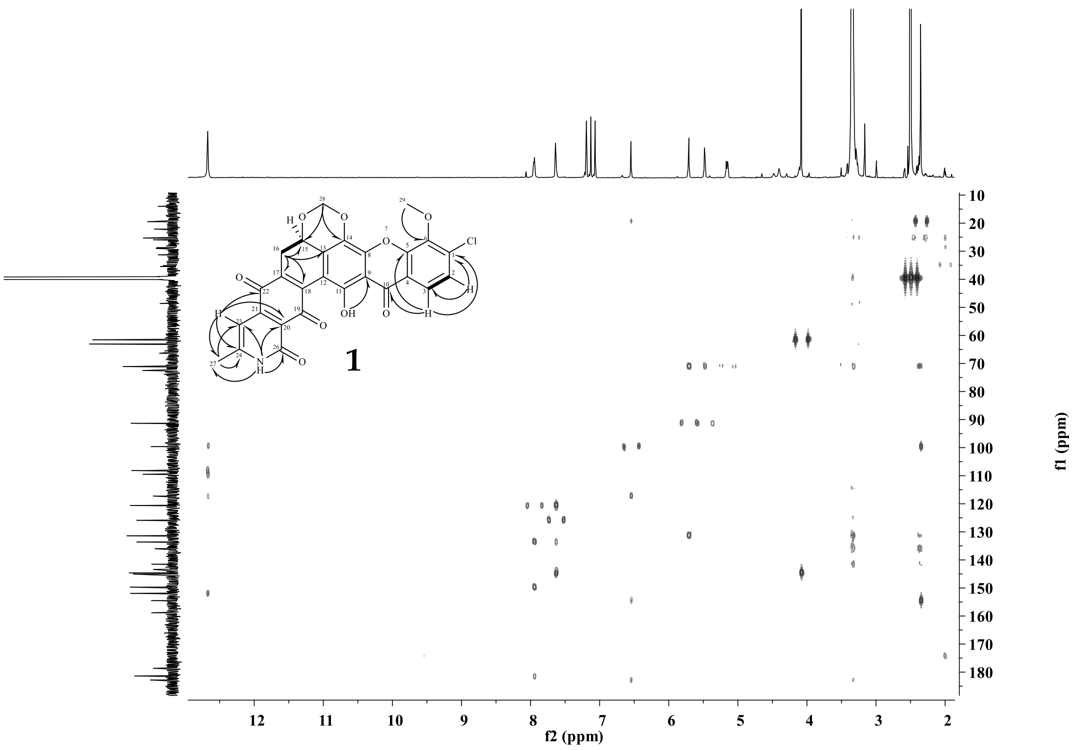

Figure S8. HMBC Spectrum of 15*R*-17,18-dehydroxantholipin (**1**).

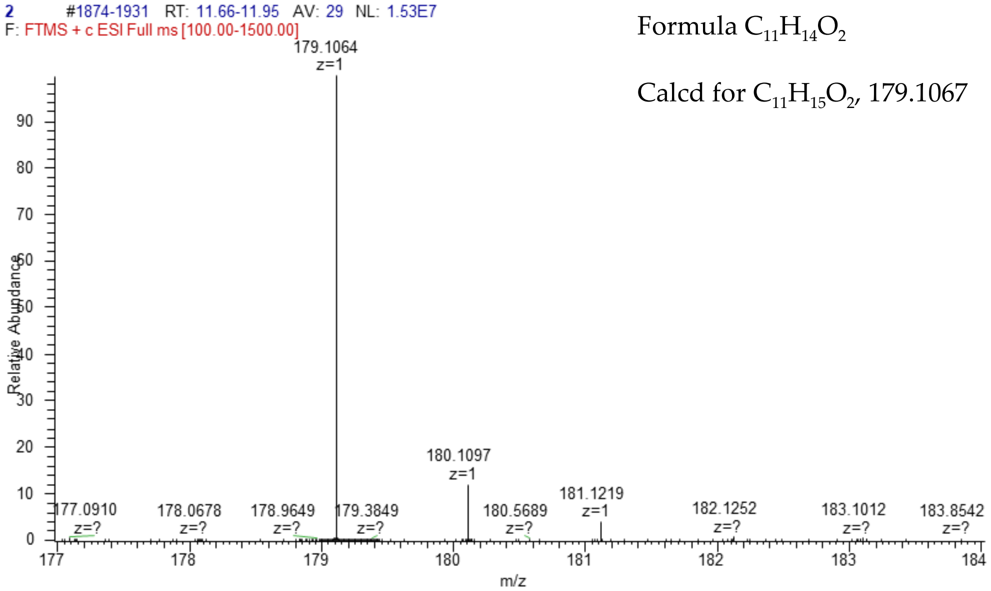

Formula $C_{11}H_{14}O_2$

Calcd for $C_{11}H_{15}O_2$, 179.1067

Figure S9. HERESIMS spectrum of (3*E*,5*E*,7*E*)-3-methyldeca-3,5,7-triene-2,9-dione (**2**).

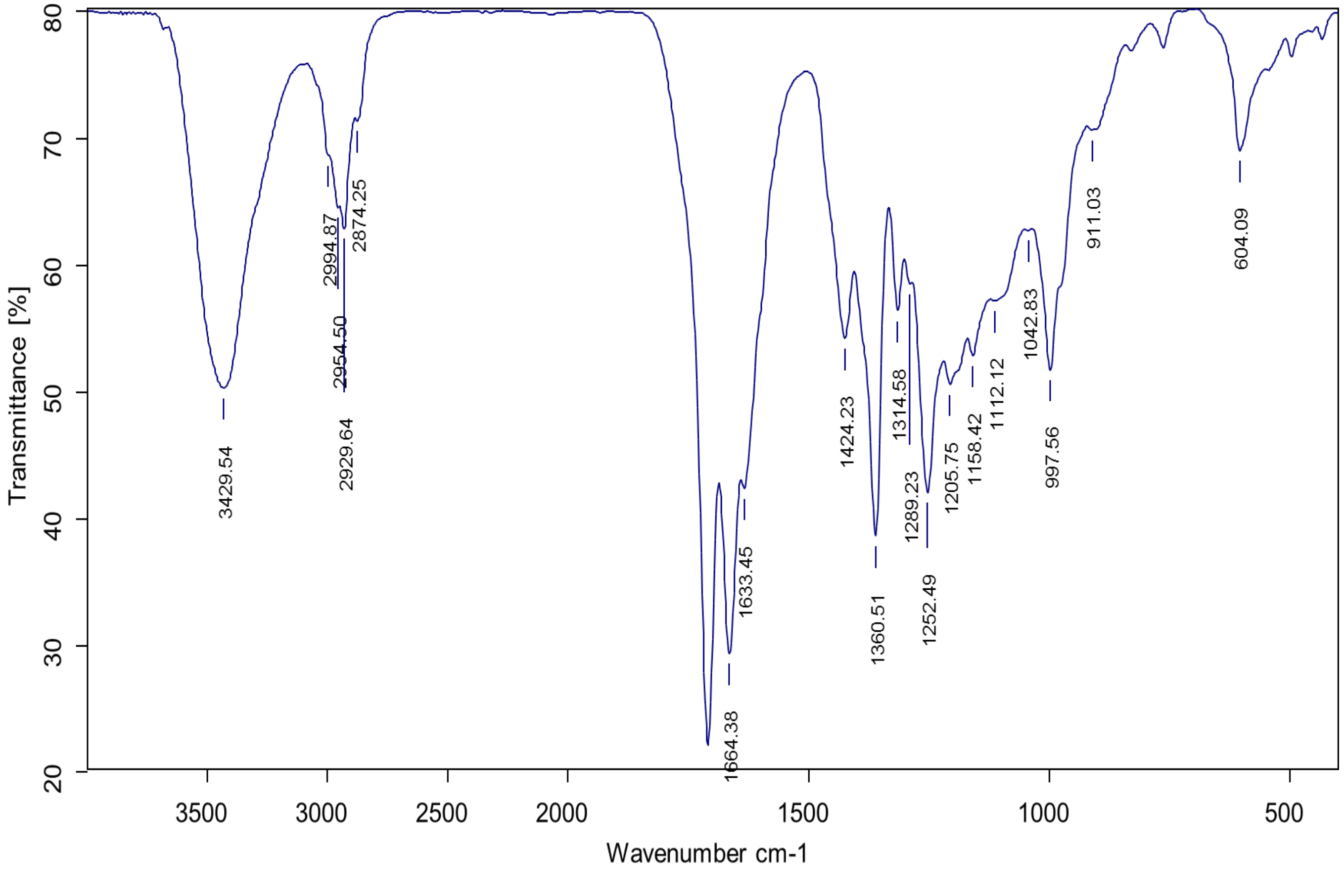

Figure S10. IR spectrum of (3*E*,5*E*,7*E*)-3-methyldeca-3,5,7-triene-2,9-dione (**2**).

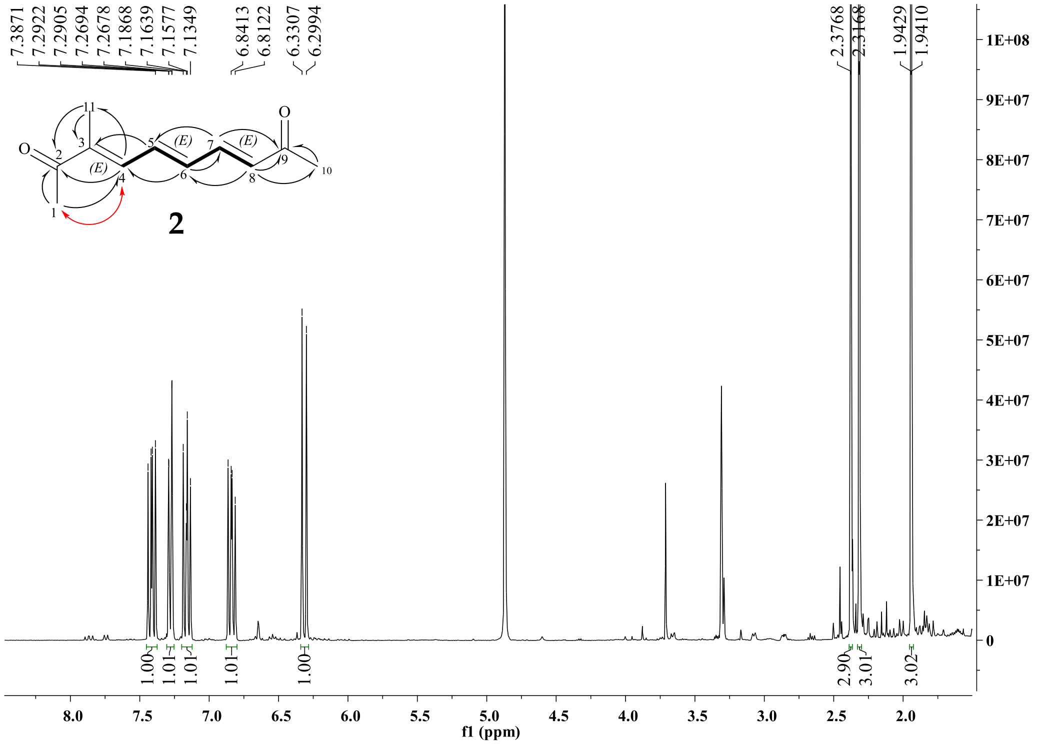

Figure S11. ^{1}H NMR (500 MHz, CD_3OD-d_4) of (3*E*,5*E*,7*E*)-3-methyldeca-3,5,7-triene-2,9-dione (**2**).

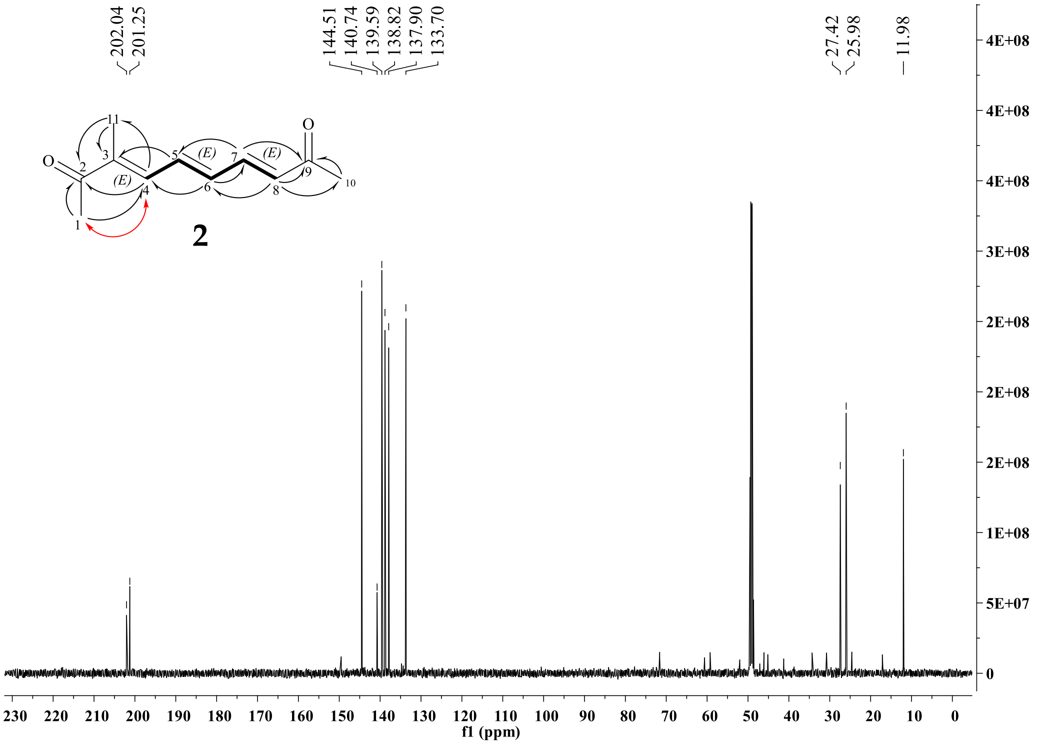

Figure S12. ^{13}C NMR (125 MHz, CD_3OD-d_4) of (3*E*,5*E*,7*E*)-3-methyldeca-3,5,7-triene-2,9-dione (**2**).

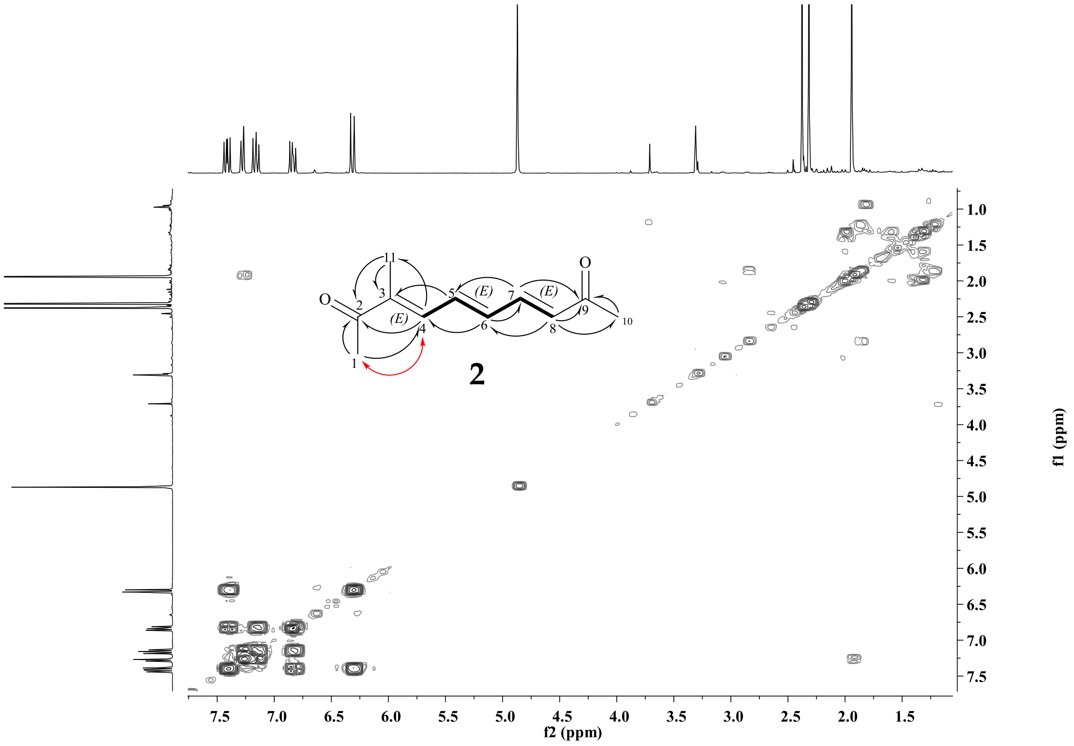

Figure S13. COSY Spectrum of (3*E*,5*E*,7*E*)-3-methyldeca-3,5,7-triene-2,9-dione (**2**).

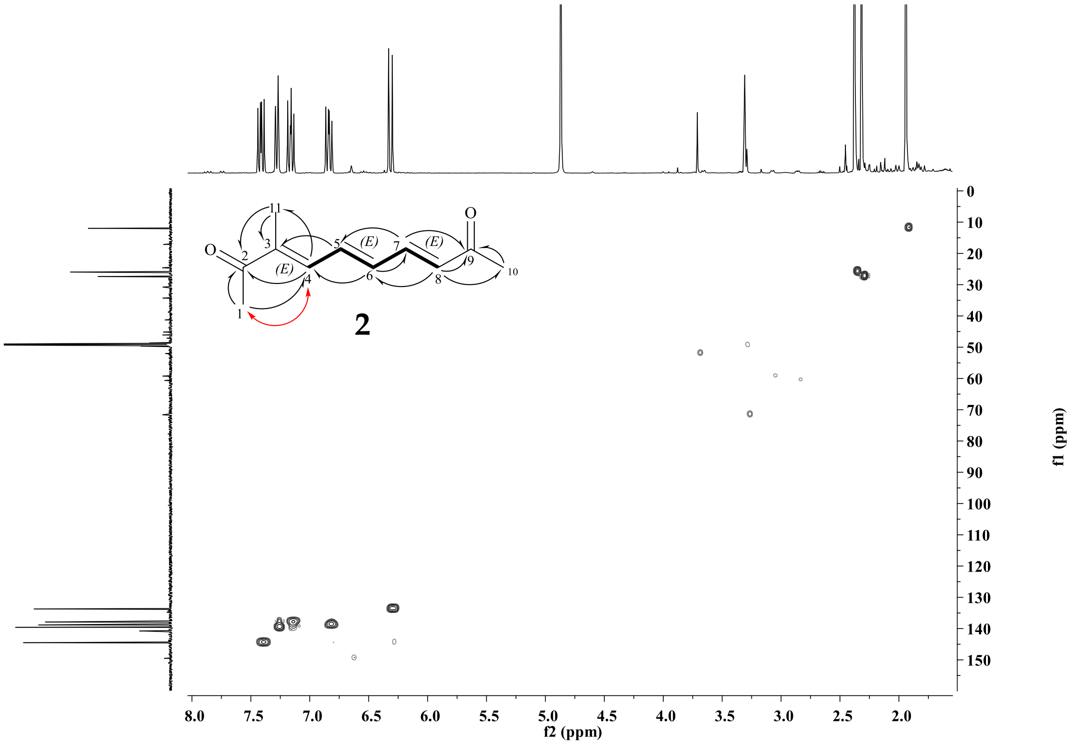

Figure S14. HSQC Spectrum of (3*E*,5*E*,7*E*)-3-methyldeca-3,5,7-triene-2,9-dione (**2**).

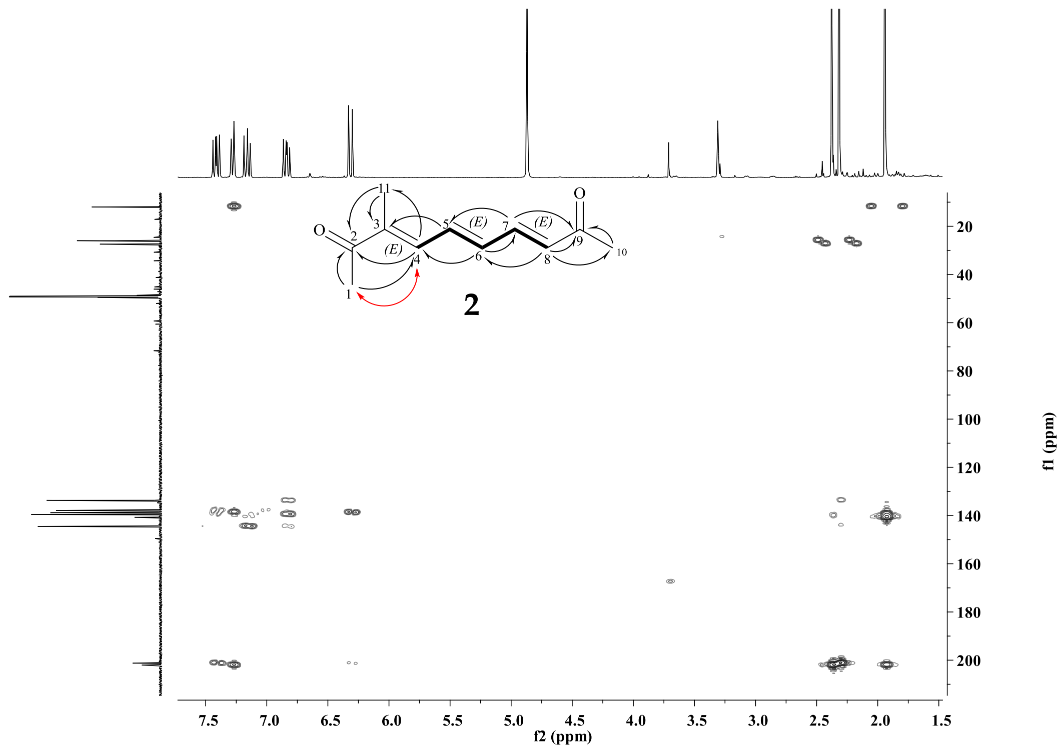

Figure S15. HMBC Spectrum of (3*E*,5*E*,7*E*)-3-methyldeca-3,5,7-triene-2,9-dione (**2**).

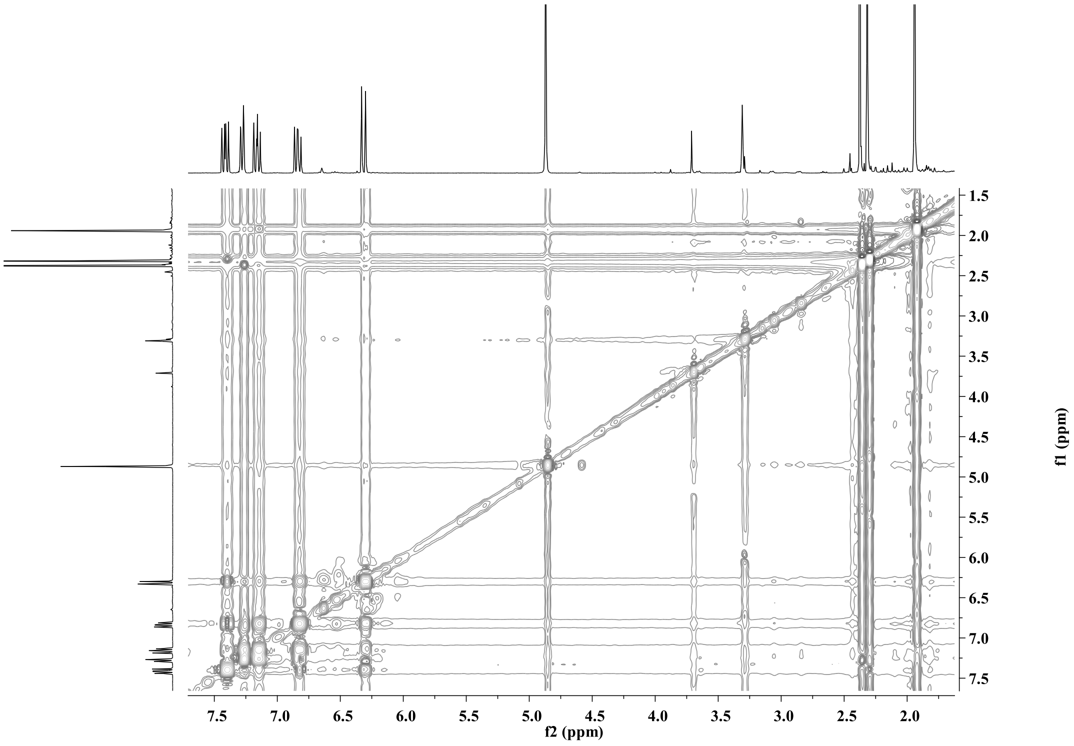

Figure S16. ROESY Spectrum of (3*E*,5*E*,7*E*)-3-methyldeca-3,5,7-triene-2,9-dione (**2**).

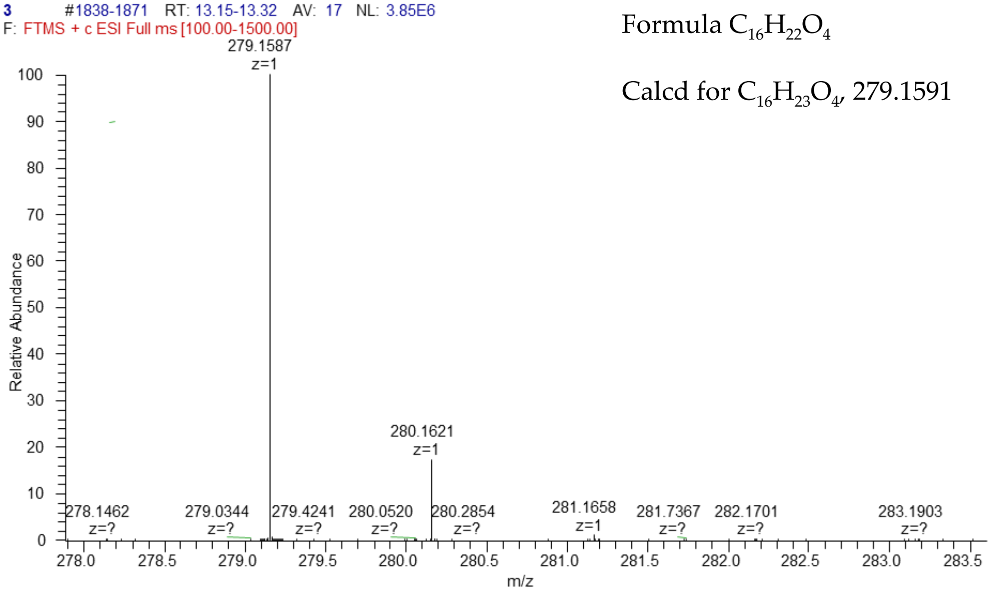

Formula $C_{16}H_{22}O_4$

Calcd for $C_{16}H_{23}O_4$, 279.1591

Figure S17. HERESIMS spectrum of qinlactone A (**3**).

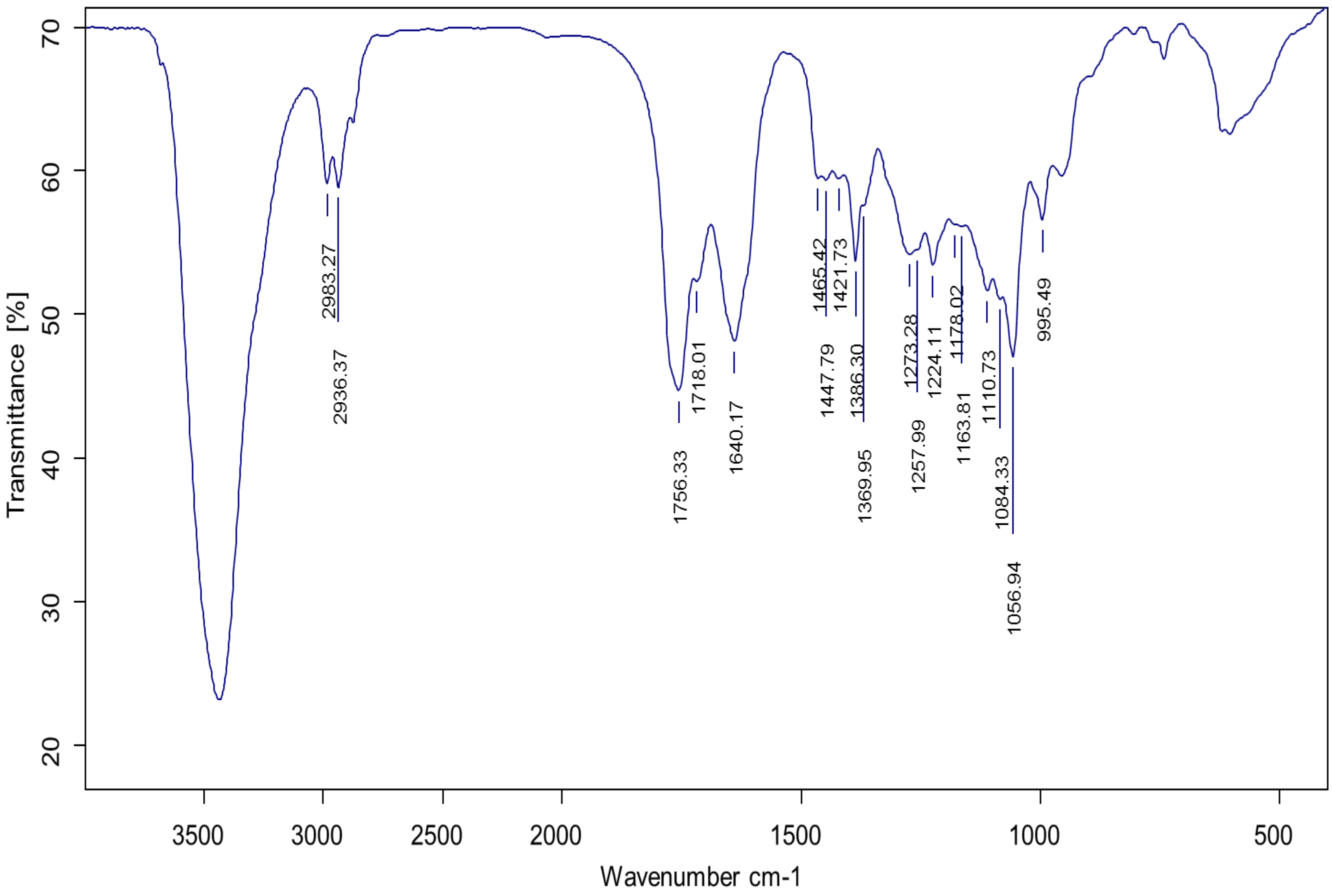

Figure S18. IR spectrum of qinlactone A (**3**).

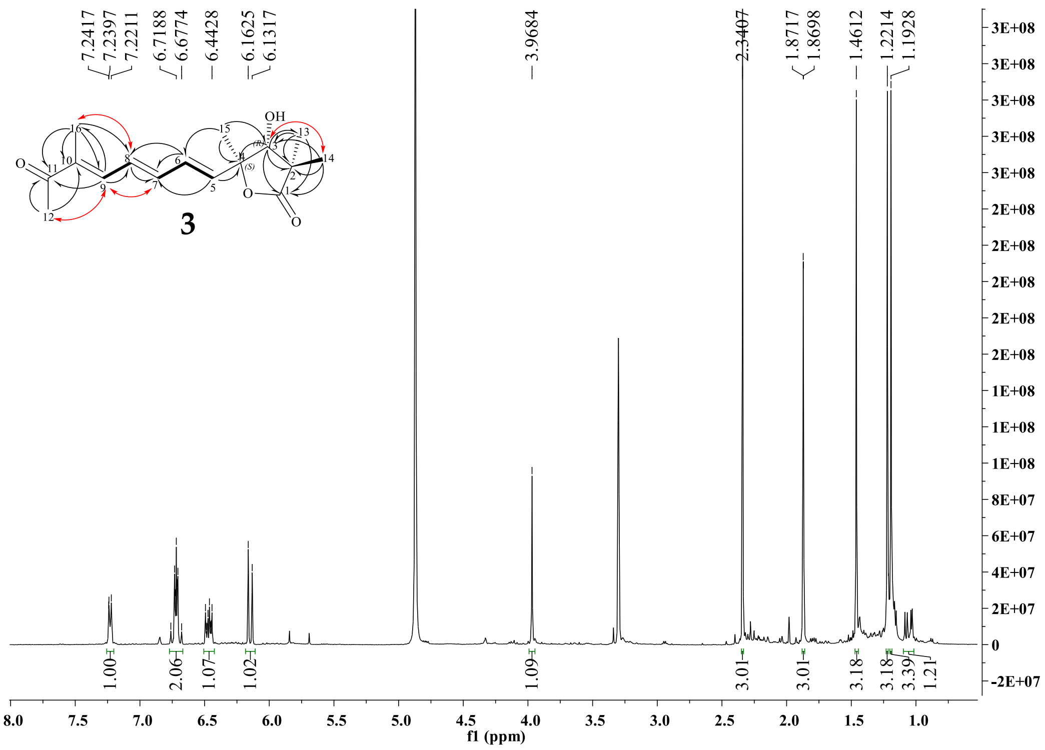

Figure S19. ^{1}H NMR Spectrum (500 MHz, CD_3OD-*d*$_4$) of qinlactone A (**3**).

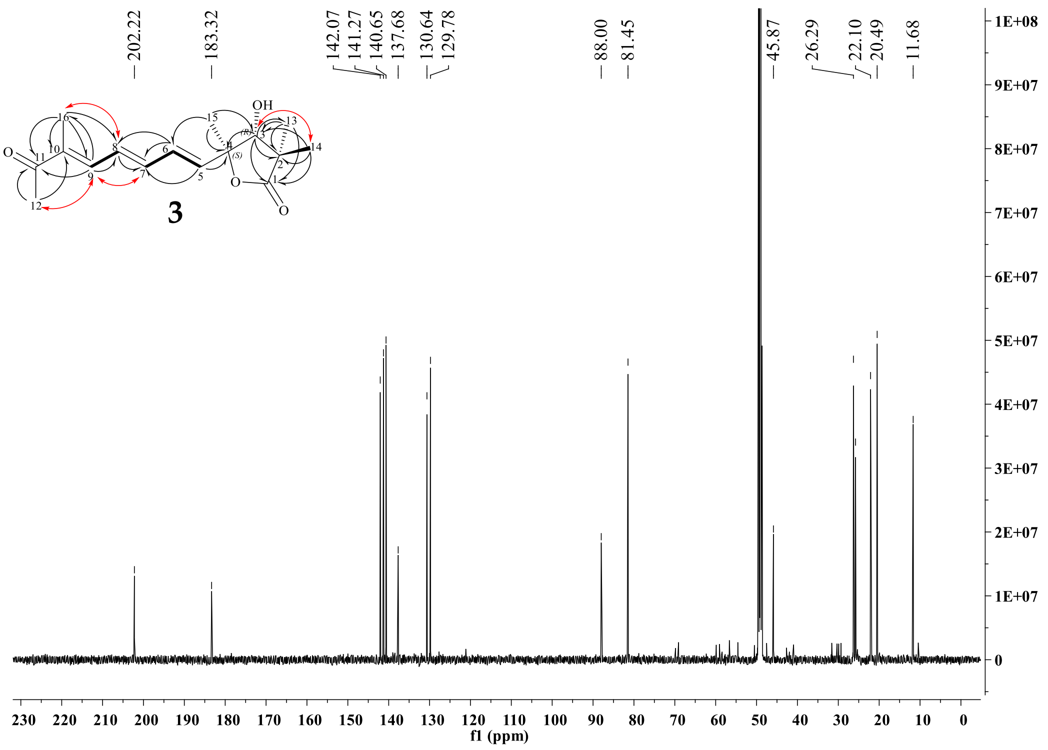

Figure S20. ^{13}C NMR Spectrum (125 MHz, CD_3OD-*d*$_4$) of qinlactone A (**3**).

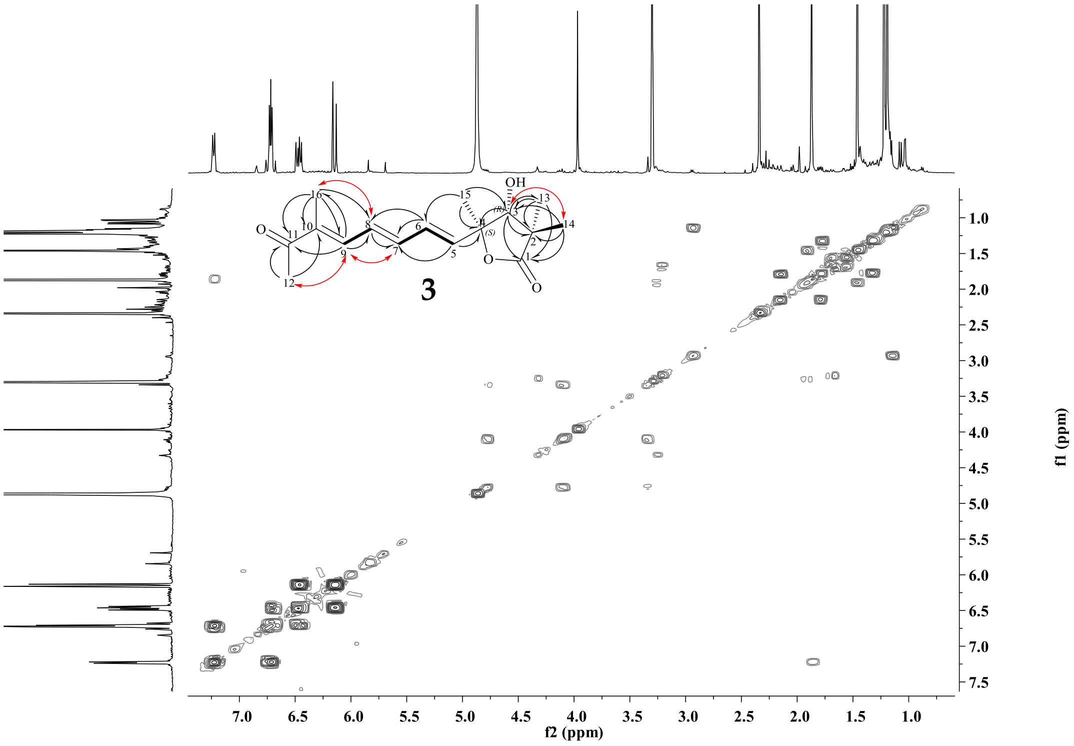

Figure S21. COSY Spectrum of qinlactone A (**3**).

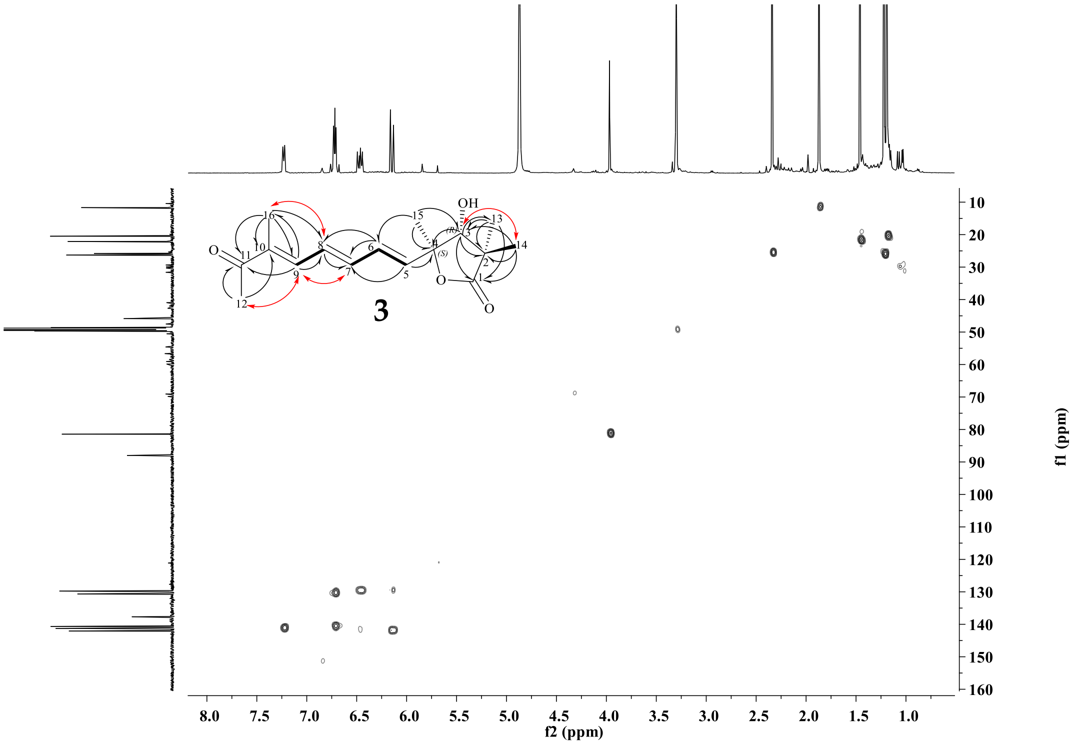

Figure S22. HSQC Spectrum of qinlactone A (**3**).

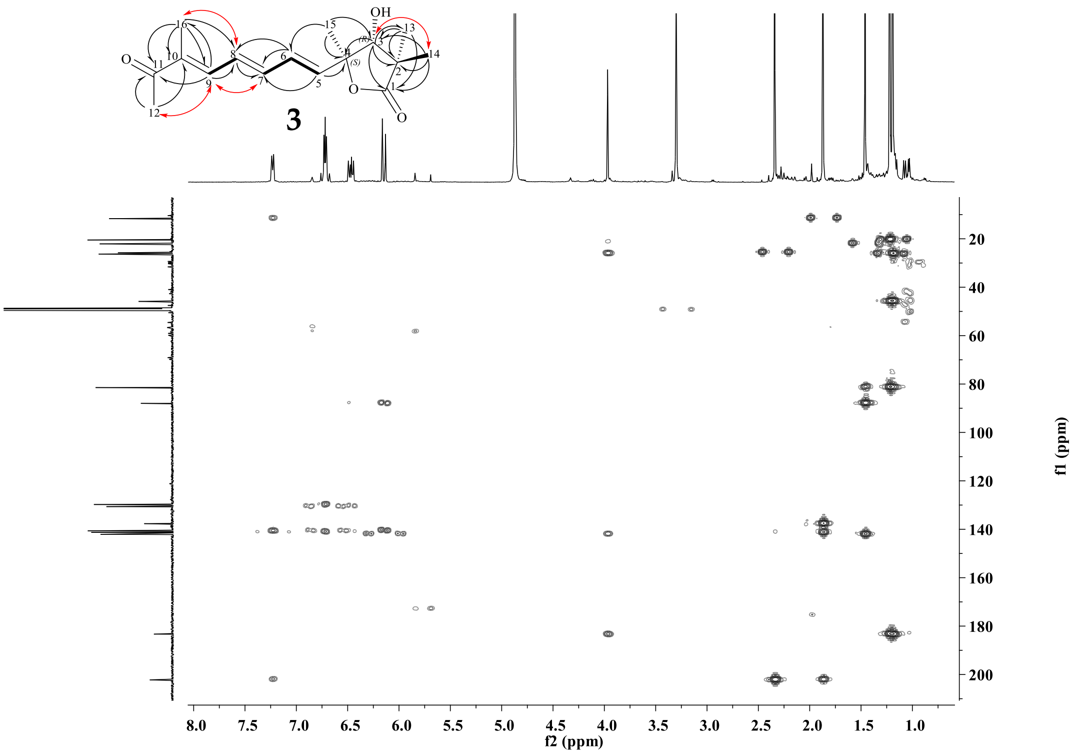

Figure S23. HMBC Spectrum of qinlactone A (**3**).

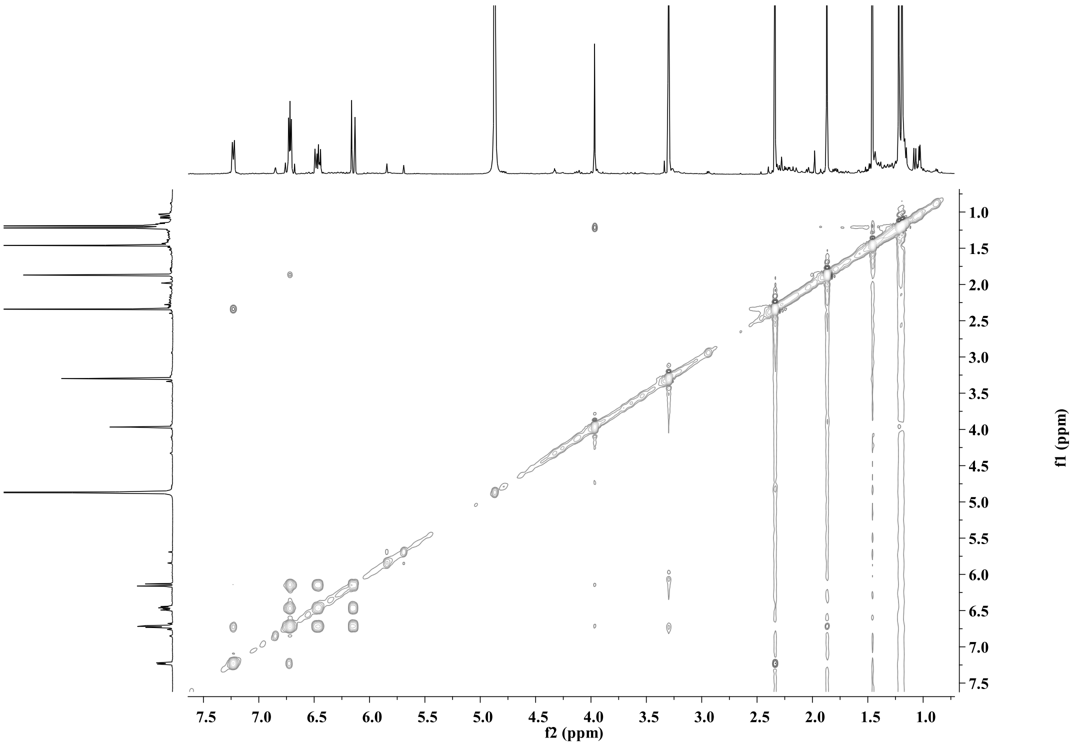

Figure S24. ROESY Spectrum of qinlactone A (**3**).

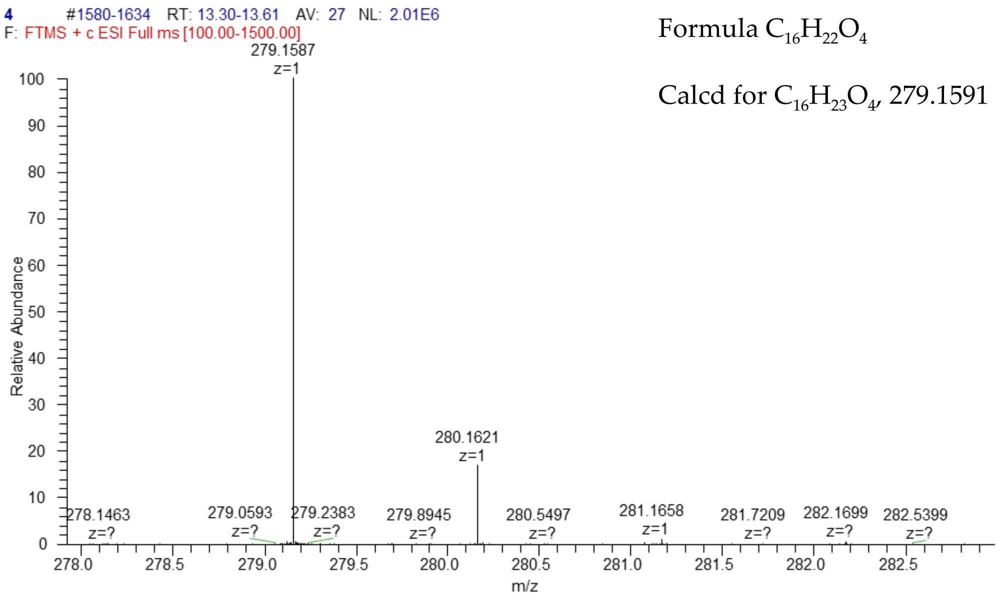

Formula $C_{16}H_{22}O_4$

Calcd for $C_{16}H_{23}O_4$, 279.1591

Figure S25. HERESIMS spectrum of qinlactone B (**4**).

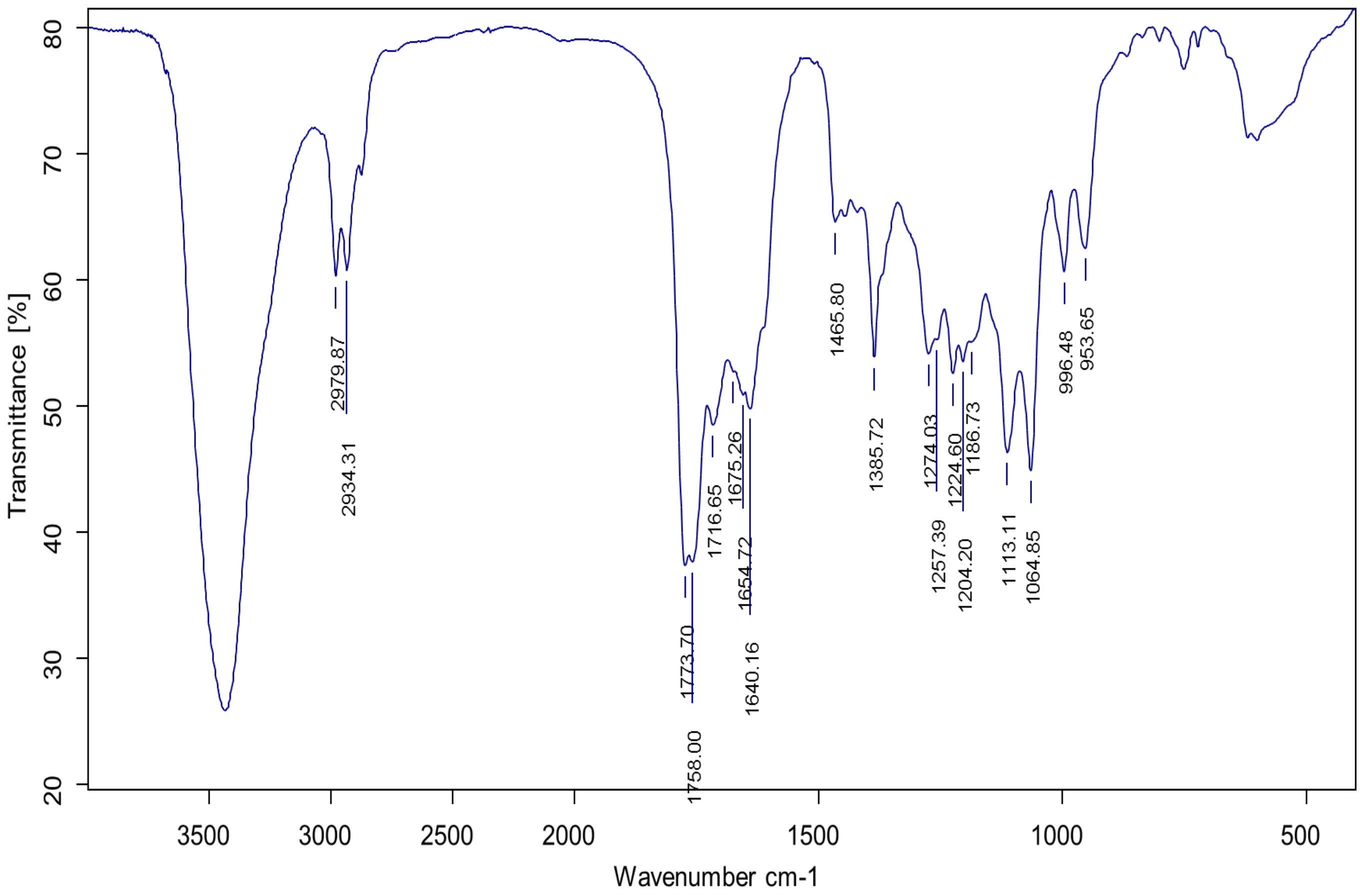

Figure S26. IR spectrum of qinlactone B (**4**).

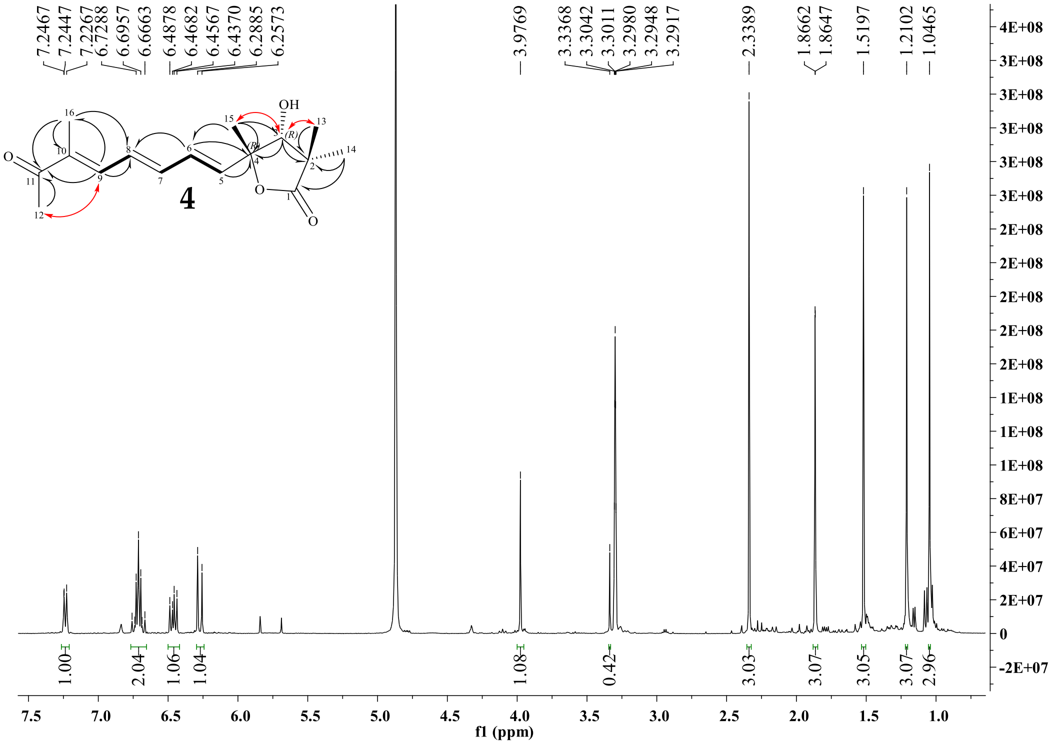

Figure S27. ^{1}H NMR Spectrum (500 MHz, CD_3OD-*d_4*) of qinlactone B (**4**).

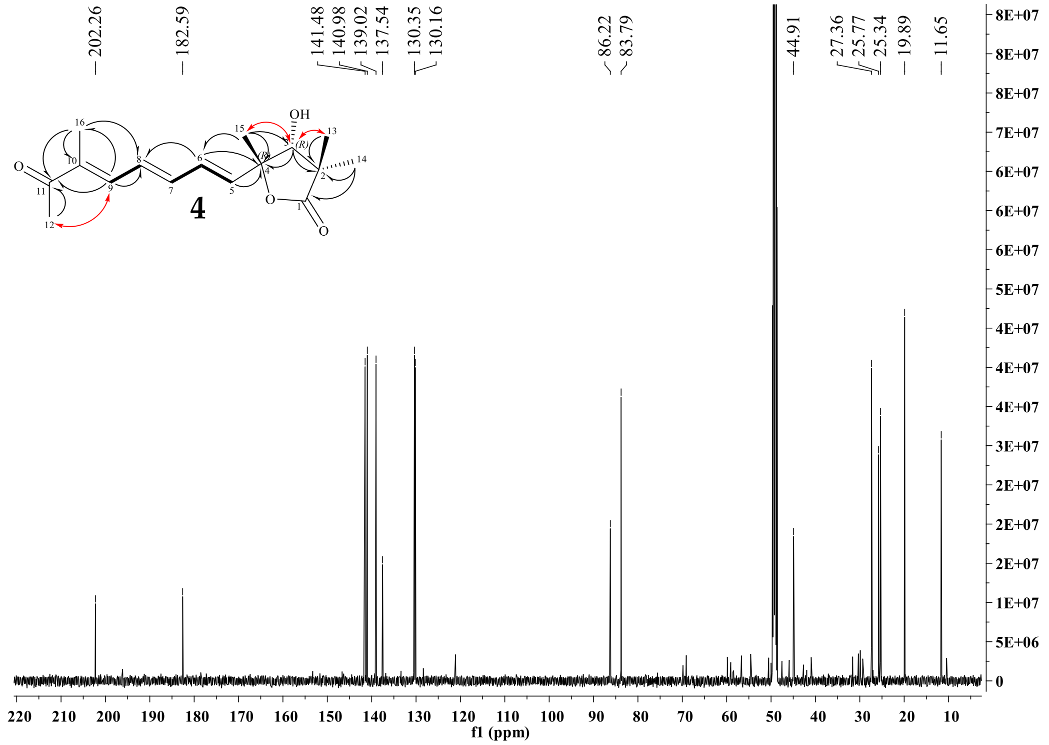

Figure S28. ^{13}C NMR Spectrum (125 MHz, CD_3OD-*d_4*) of qinlactone B (**4**).

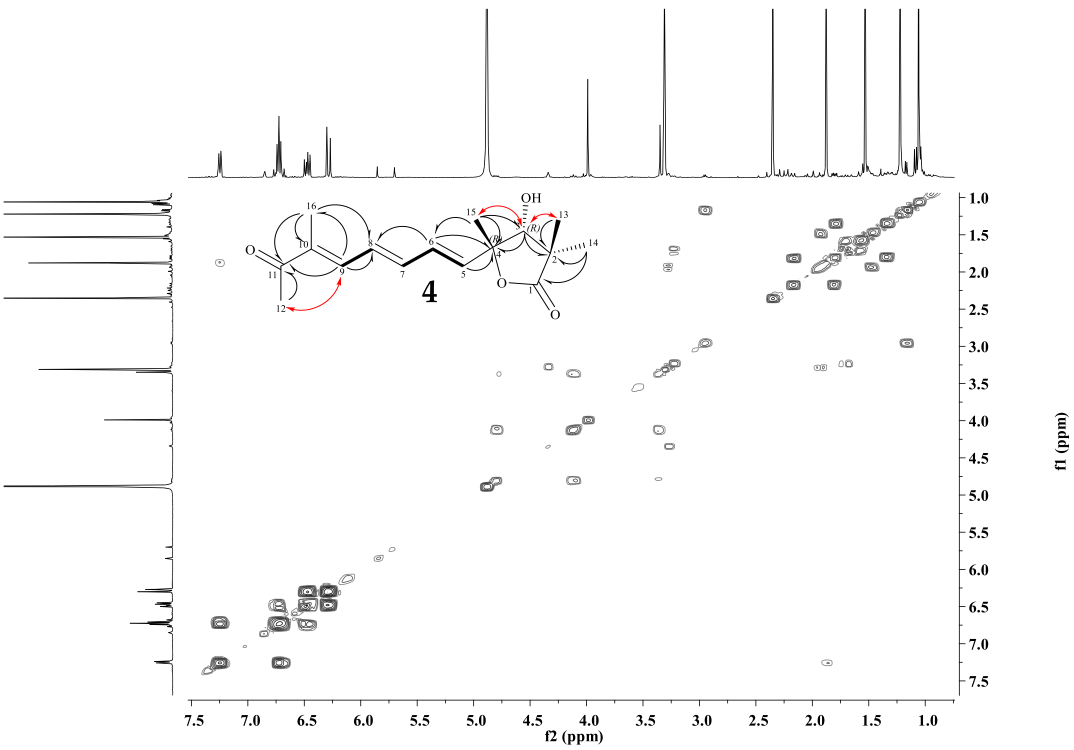

Figure S29. COSY Spectrum of qinlactone B (**4**).

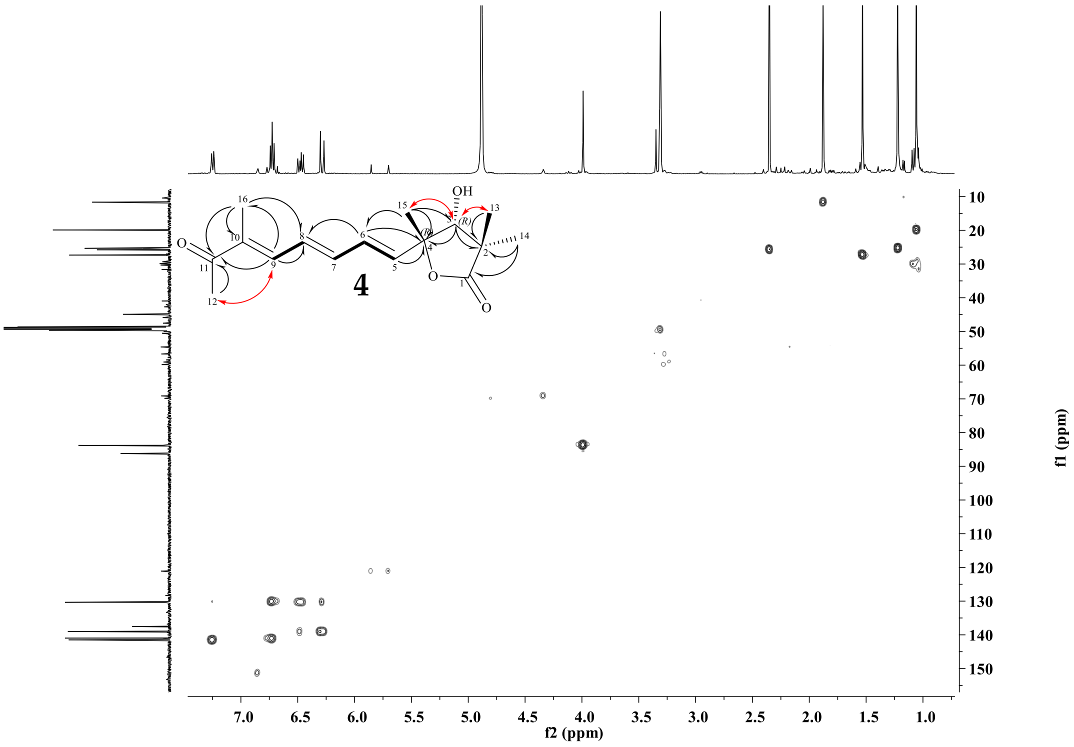

Figure S30. HSQC Spectrum of qinlactone B (**4**).

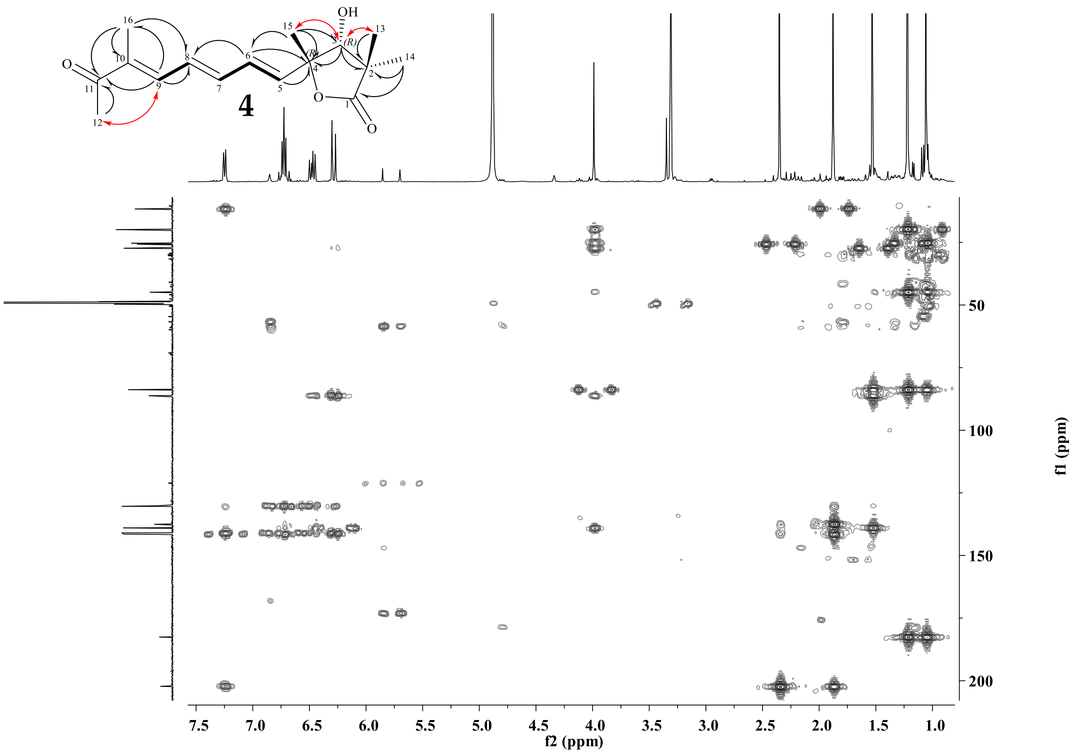

Figure S31. HMBC Spectrum of qinlactone B (**4**).

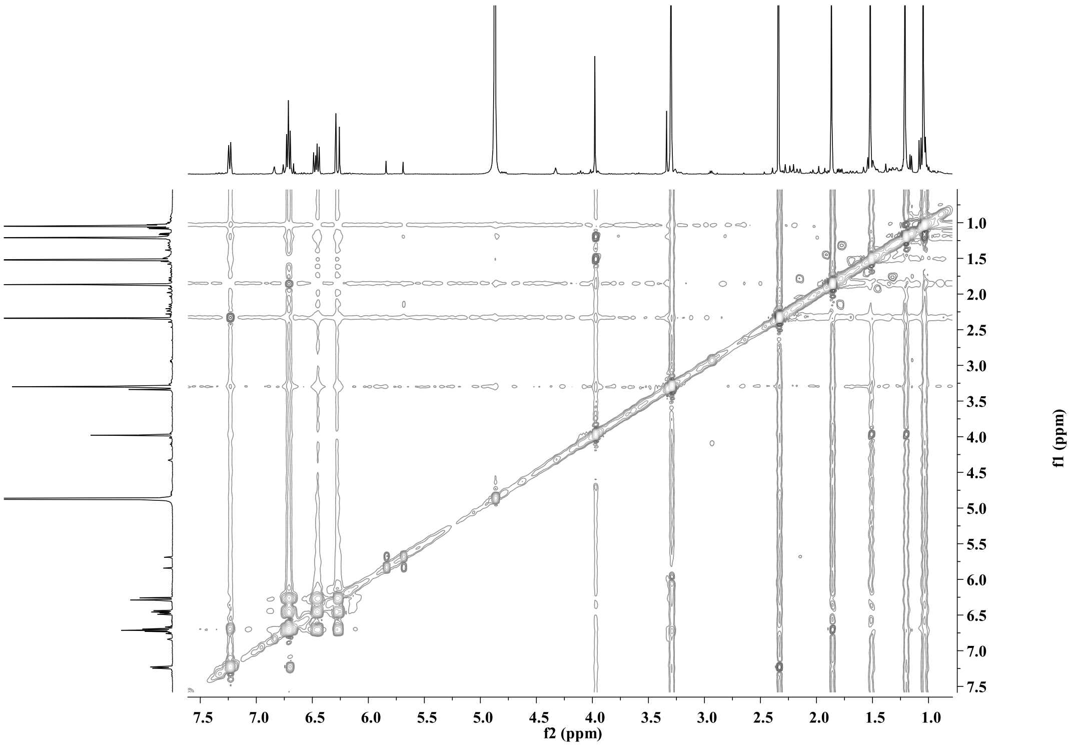

Figure S32. ROESY Spectrum of qinlactone B (**4**).

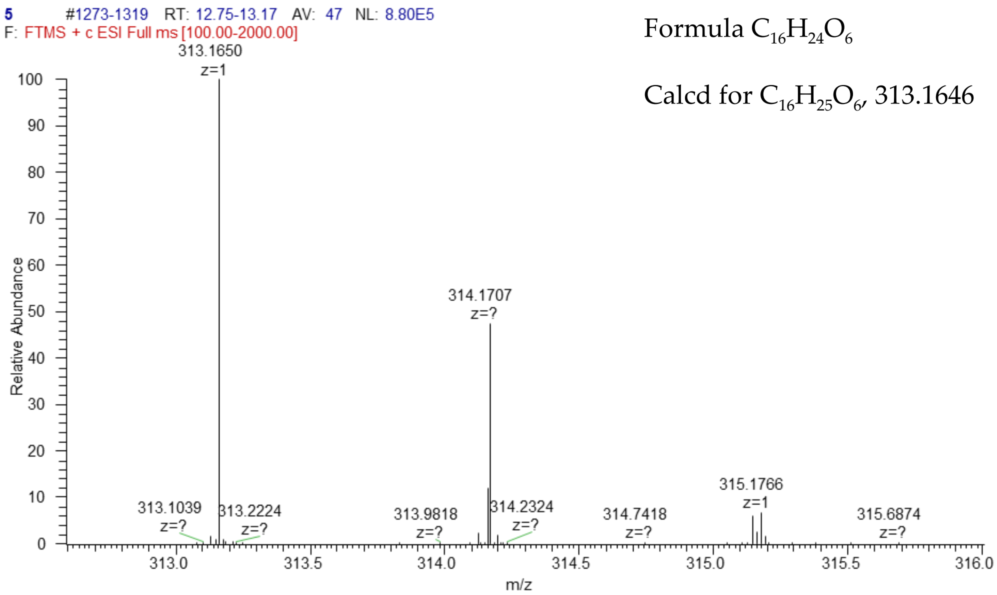

Formula $C_{16}H_{24}O_6$

Calcd for $C_{16}H_{25}O_6$, 313.1646

Figure S33. HERESIMS spectrum of qinlactone C (**5**).

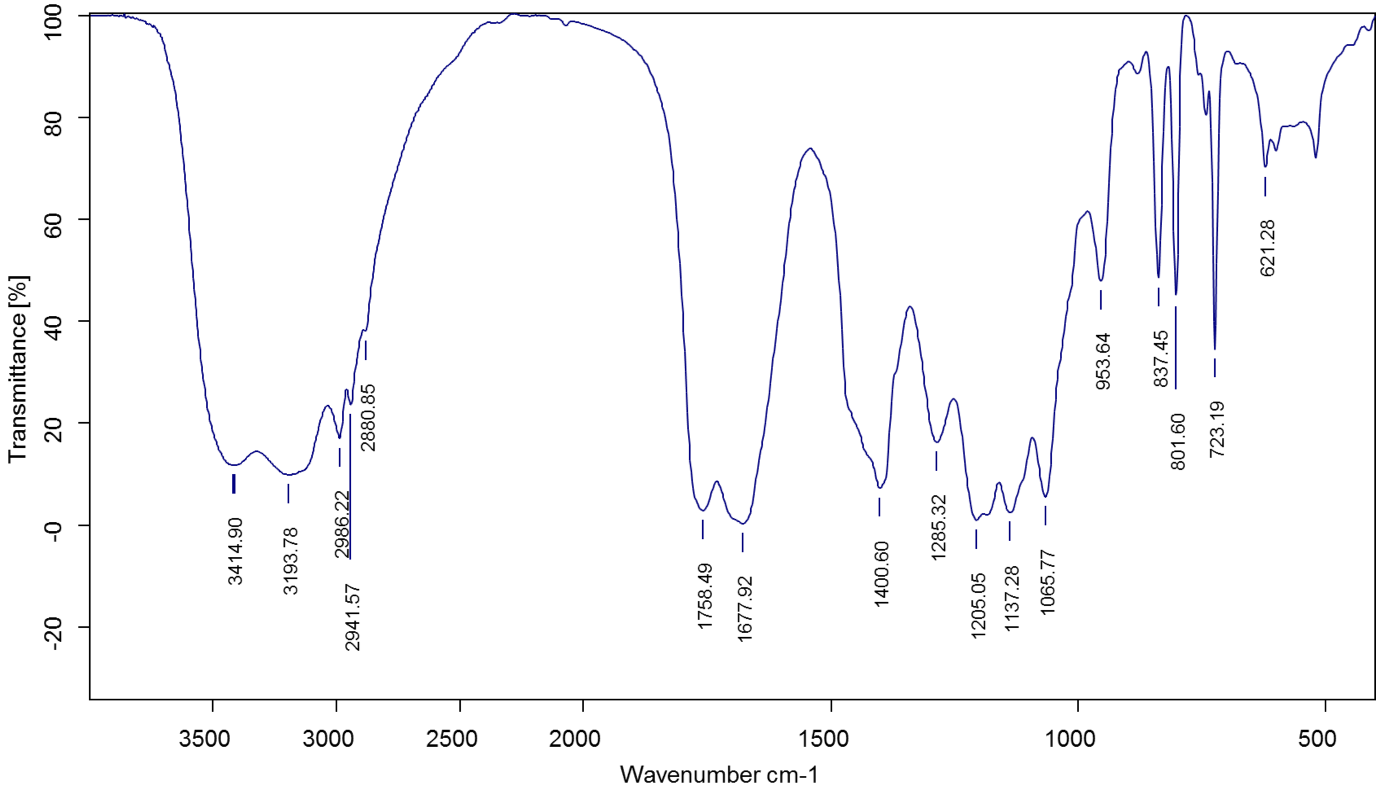

Figure S34. IR spectrum of qinlactone C (**5**).

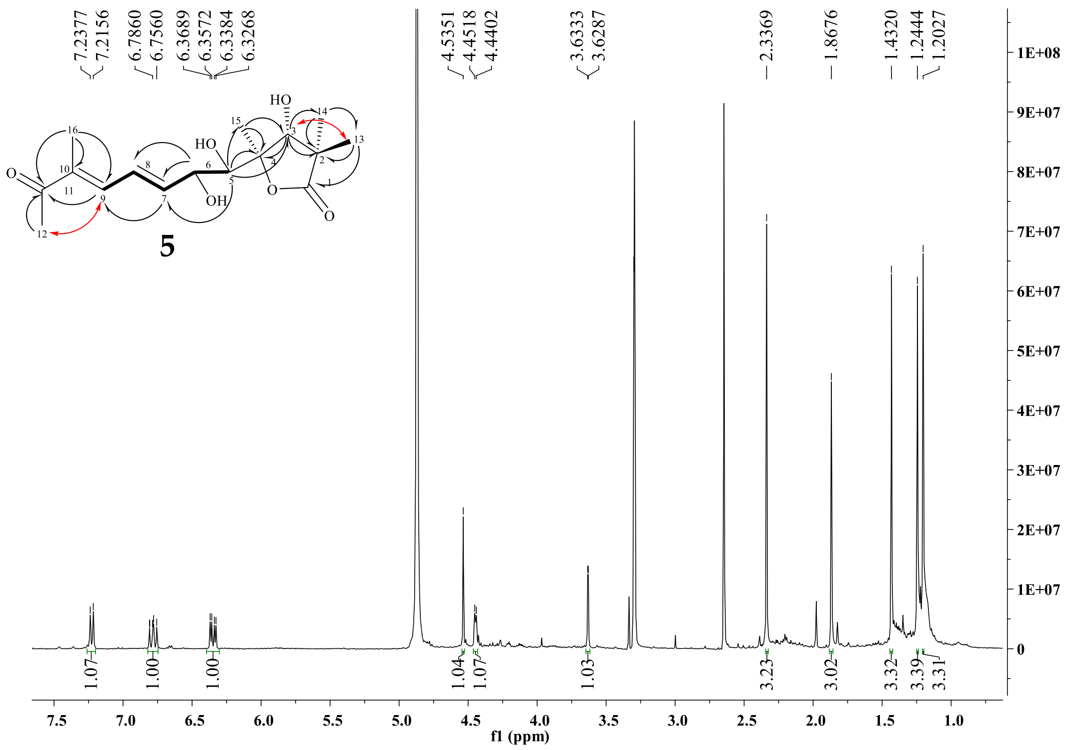

Figure S35. ^{1}H NMR Spectrum (500 MHz, CD_3OD-*d*$_4$) of qinlactone C (**5**).

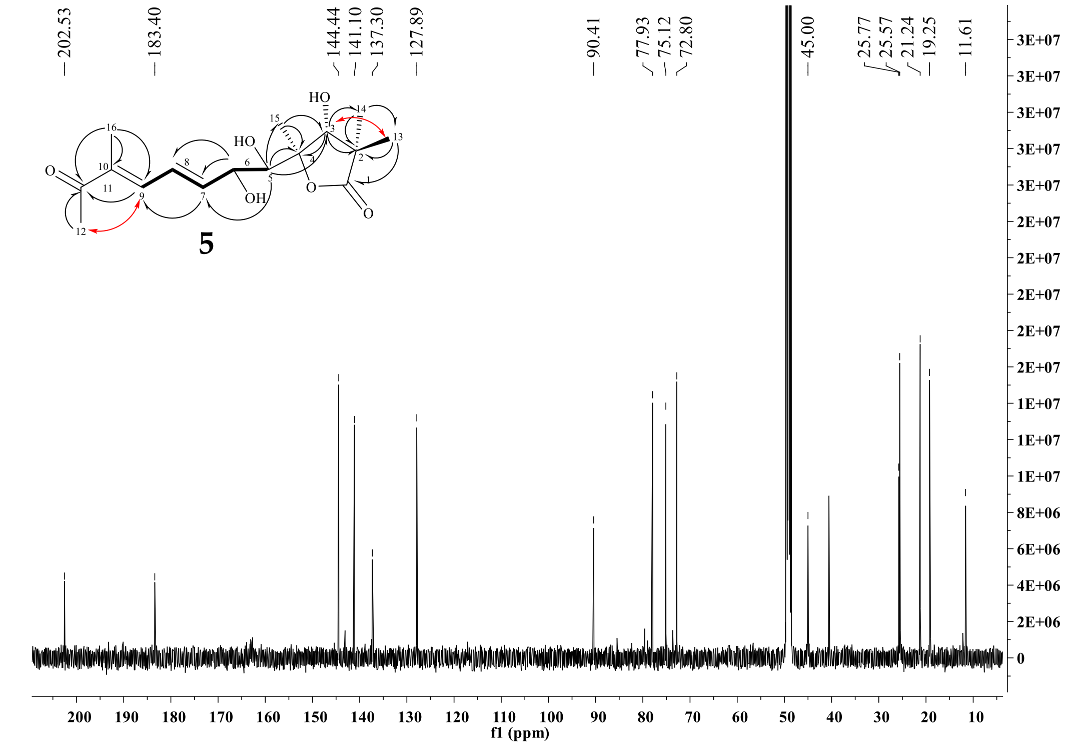

Figure S36. ^{13}C NMR Spectrum (125 MHz, CD_3OD-*d*$_4$) of qinlactone C (**5**).

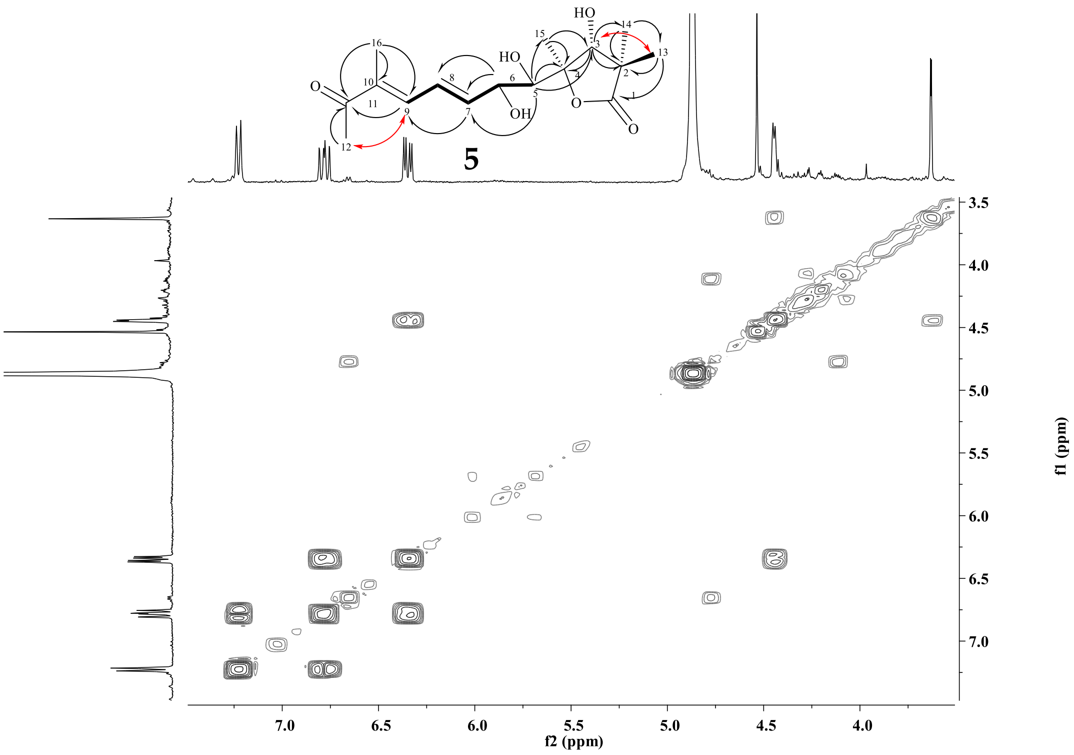

Figure S37. COSY Spectrum of qinlactone C (**5**).

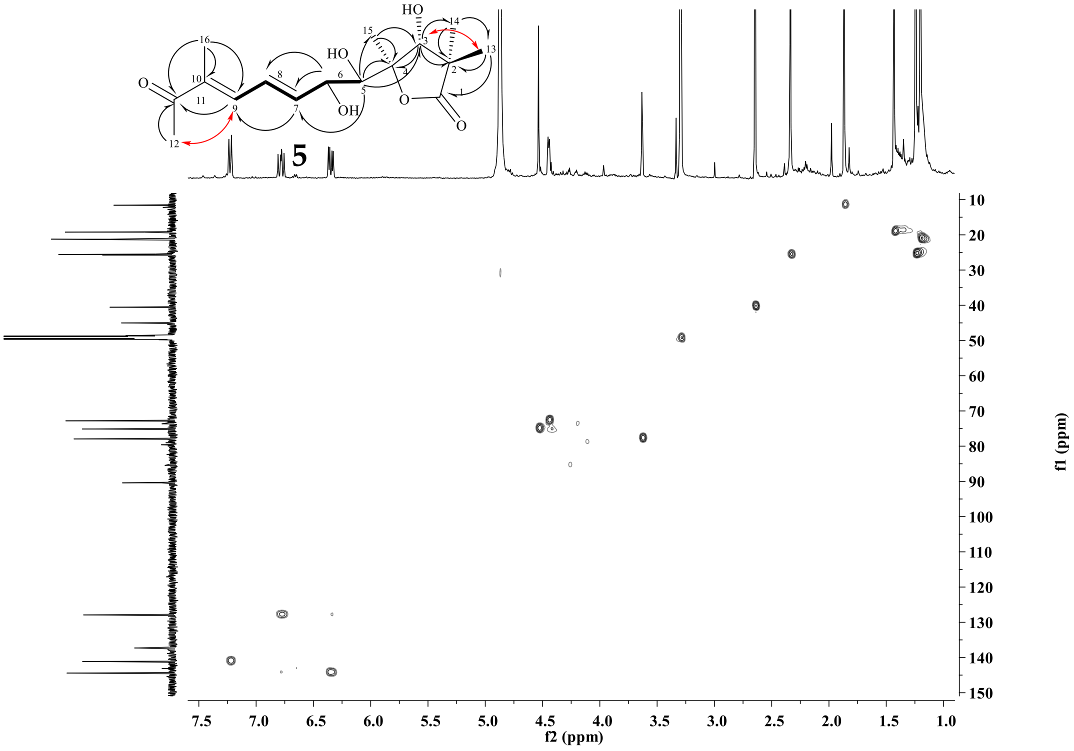

Figure S38. HSQC Spectrum of qinlactone C (**5**).

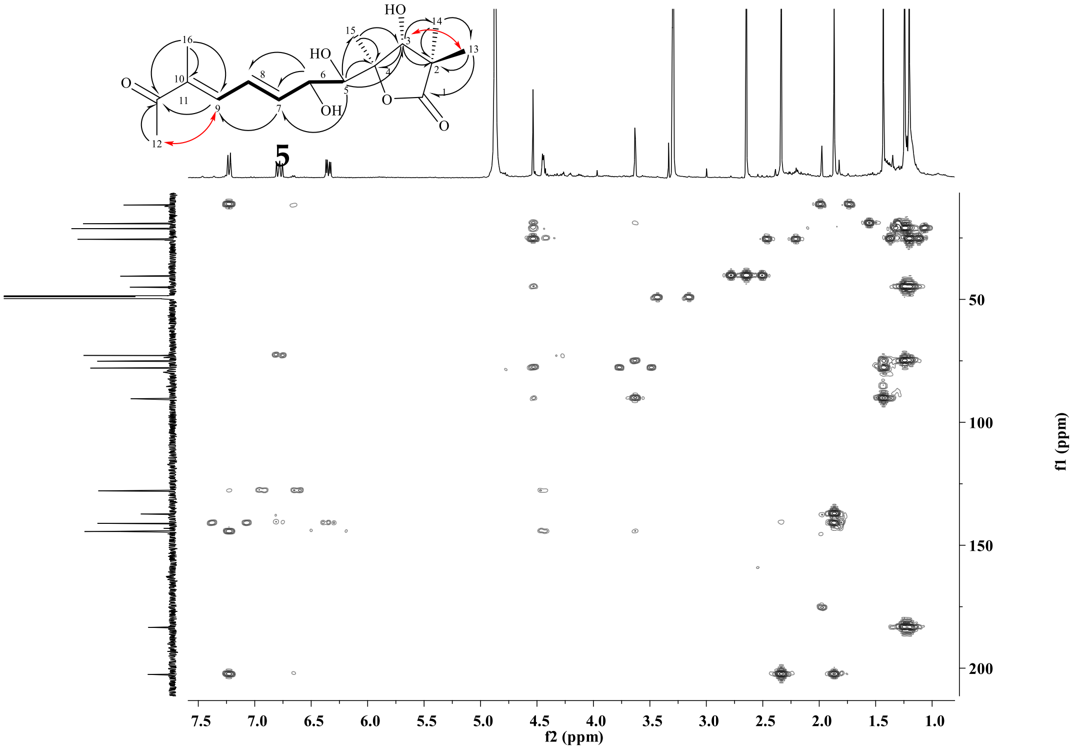

Figure S39. HMBC Spectrum of qinlactone C (**5**).

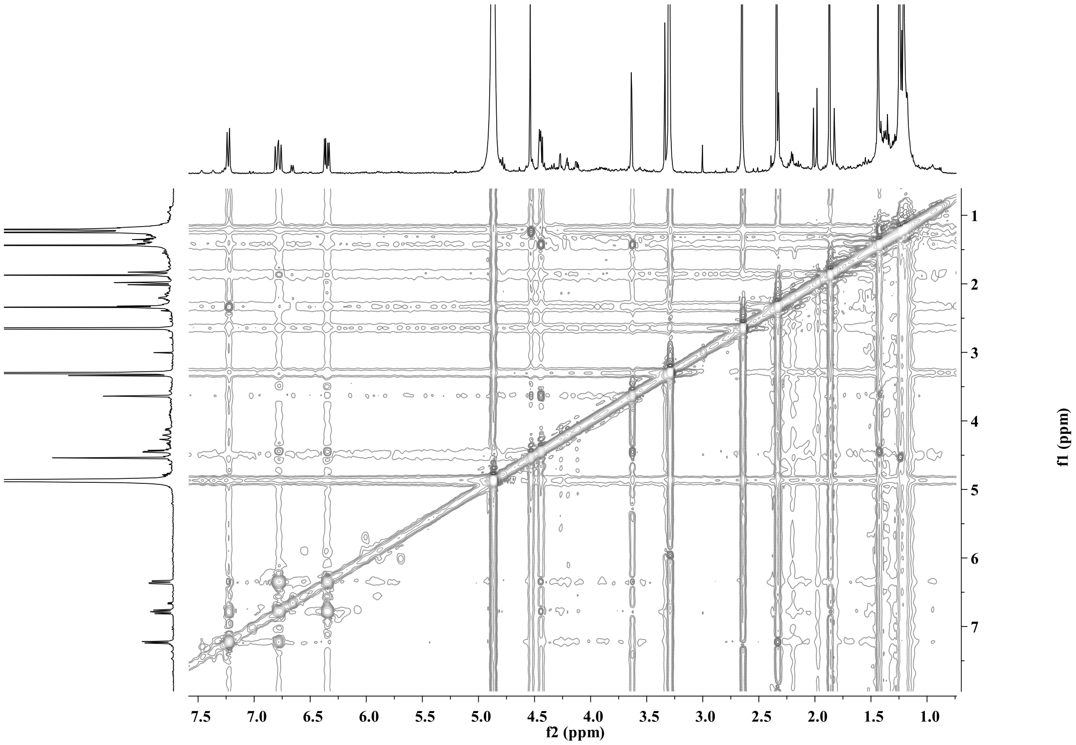

Figure S40. ROESY Spectrum of qinlactone C (**5**).

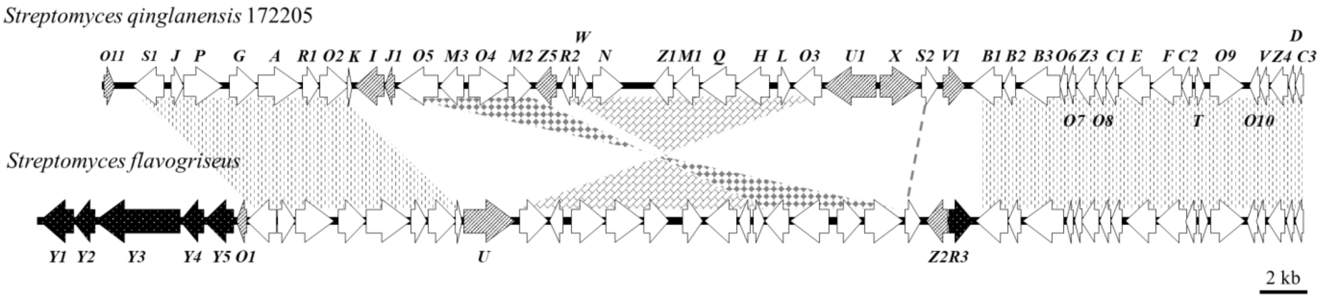

Figure S41. Comparison of genetic organization of *xan*$_q$ in strain 172205 and *xan* in *S. flavogriseus*.
